# Interpreting molecular similarity between patients as a determinant of disease comorbidity relationships

**DOI:** 10.1038/s41467-020-16540-x

**Published:** 2020-06-05

**Authors:** Jon Sánchez-Valle, Héctor Tejero, José María Fernández, David Juan, Beatriz Urda-García, Salvador Capella-Gutiérrez, Fátima Al-Shahrour, Rafael Tabarés-Seisdedos, Anaïs Baudot, Vera Pancaldi, Alfonso Valencia

**Affiliations:** 1 Barcelona Supercomputing Center (BSC), 08034 Barcelona, Spain; 2 Structural Biology Program, Spanish National Cancer Research Center (CNIO), 28029 Madrid, Spain; 3 Coordination Node, Spanish National Bioinformatics Institute, ELIXIR-Spain (INB, ELIXIR-ES), Madrid, Spain; 4 Institute of Evolutionary Biology (UPF-CSIC), PRBB, Dr. Aiguader 88, 08003 Barcelona, Spain; 5 Department of Medicine, University of Valencia, CIBERSAM, INCLIVA, 46010 Valencia, Spain; 6 Aix Marseille Univ, INSERM, MMG, Marseille, France; 7 CNRS, Marseille, France; 8 Centre de Recherches en Cancérologie de Toulouse (CRCT), UMR1037 Inserm, ERL5294 CNRS, 2 Avenue Hubert Curien, 31037 Toulouse, France; 9 University Paul Sabatier III, Toulouse, France; 10 ICREA, 08010 Barcelona, Spain

**Keywords:** Cancer, Computational biology and bioinformatics, Genetics, Gene expression

## Abstract

Comorbidity is a medical condition attracting increasing attention in healthcare and biomedical research. Little is known about the involvement of potential molecular factors leading to the emergence of a specific disease in patients affected by other conditions. We present here a disease interaction network inferred from similarities between patients’ molecular profiles, which significantly recapitulates epidemiologically documented comorbidities. Furthermore, we identify disease patient-subgroups that present different molecular similarities with other diseases, some of them opposing the general tendencies observed at the disease level. Analyzing the generated patient-subgroup network, we identify genes involved in such relations, together with drugs whose effects are potentially associated with the observed comorbidities. All the obtained associations are available at the disease PERCEPTION portal (http://disease-perception.bsc.es).

## Abstract (web summary)

Disease comorbidity is attracting increasing attention, but the involvement of molecular factors in forecasting risk of a disease in the presence of other diseases is poorly understood. Here the authors build a disease interaction network based on gene expression profile and discover new comorbidity relationships in patient subgroups.

## Introduction

Comorbidity is the altered risk for patients to develop a second disease when they are already suffering from a specific one. Comorbidity incidence increases with age and has a high impact on life expectancy, which decreases considerably in the presence of simultaneous diseases^1^, as is commonly observed in ageing populations^2^. Additionally, the presence of comorbid conditions in patients has a high economic impact as shown, for example, by the 150% increase in the cost associated with diabetes for people who are also affected by heart diseases^3^. Thus, controlling patient-specific risks of future comorbidities could increase life expectancy and reduce public health expenditure^4^.

Previous observations on schizophrenic patients regarding the risk of developing lung cancer (decreased in both schizophrenic patients and their relatives^5^) suggested to us that there might be a potential molecular basis of comorbidity relations. Supporting this hypothesis, tens of disease−disease interaction networks have been published since 2007^6^, using a variety of data, such as gene expression profiles^7^, combinations of disease genes and protein−protein interaction networks^8^, miRNA expression^9^, the microbiome^10^, medical claims^11^, medical records^12^, human symptoms^13^, insurance claims^14^, or mixed information^15^. Importantly, several studies pointed out that known comorbid diseases, despite not sharing a single disease-gene, fell into the same neighborhood of the interactome, providing insights into the molecular bases of their comorbidity relation^8,16,17^.

Regarding the analysis of electronic health records, Beck et al.^12^ consider that patients with the same disease might present different risks of developing secondary diseases based on their temporally resolved medical records. We hypothesize that this can be a consequence of the existence of different clinical phenotypes within heterogeneous medical conditions, which can lead to specific comorbidity risks. The existence of disease subtypes is well recognized in cancer, but it has also been observed for instance for chronic obstructive pulmonary disease, where four different phenotypes with clinical relevance and therapeutic repercussion have been identified^18^. Traditionally, gene expression data have been widely used to identify disease subtypes, like in the case of breast cancer^19^ and Crohn’s disease^20^.

Here, we make use of this well-accepted gene-expression-based disease classification to explore the molecular bases of comorbidity, subdividing diseases based on patient-similarities, and to define personalized comorbidity risks. We develop a patient similarity network of more than 6000 patients affected by 132 diseases, including 15 of the top 20 leading causes of death worldwide in 2015 ^21^.

**Fig. 1:**
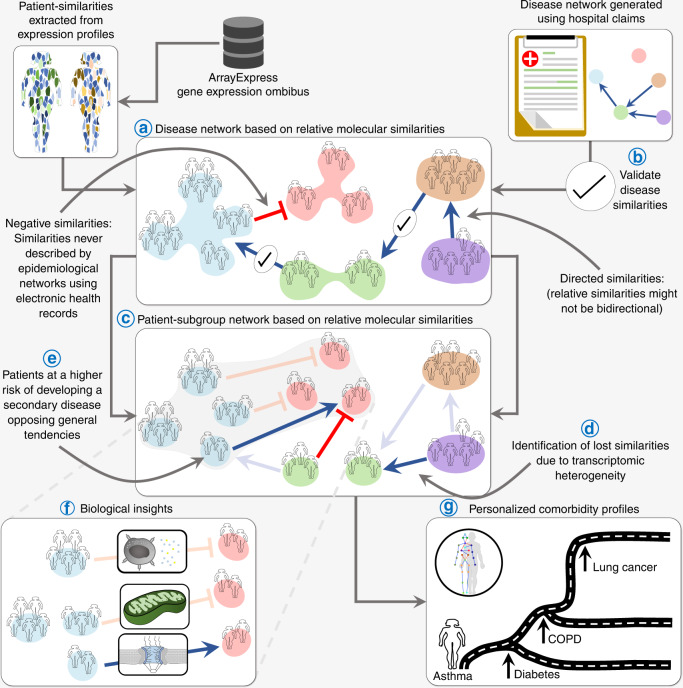
Main objectives. Throughout the study, we generate a Disease Molecular Similarity Network containing directed positive and negative Relative Molecular Similarity (pRMS and nRMS) interactions (**a**). pRMS are validated using epidemiological networks extracted from medical and hospital claims (**b**). We stratify patients into subgroups and calculate subgroup-specific relative molecular similarities (**c**), identifying similarities that were not recovered when working at the disease level (**d**), including some interactions opposing general trends (**e**). We propose biological processes potentially involved in subgroup-similarities (**f**). Finally, for each single patient, we provide a ranked list of most similar to less similar diseases based on transcriptomics information (**g**).

We calculate differential expression profiles for patients affected with different diseases, and consider how similarities between these patients’ profiles could be related to comorbidity relations between diseases (Fig. 1a). Interestingly, many of the recovered relations significantly match disease co-occurrences previously characterized in large populations by epidemiology^11,22^ (Fig. 1b). Indeed, this network confirms our previous observations of the existence of molecular mechanisms potentially underlying comorbidity relationships in the specific case of central nervous system disorders and cancers^23,24^. Additionally, we identify distinct patient-subgroups with specific relative molecular similarities within diseases (Fig. 1c), sometimes even opposing the relations observed at the global disease level (Fig. 1e), i.e. two different subgroups of patients with the same disease have different potential relations (risks) with two different diseases. Finally, we calculate relative molecular similarities between each single patient and the analyzed diseases, obtaining a ranked list of the most molecularly similar diseases (Fig. 1g).

## Results

### Transcriptomics-based Disease Molecular Similarity Network

**Fig. 2:**
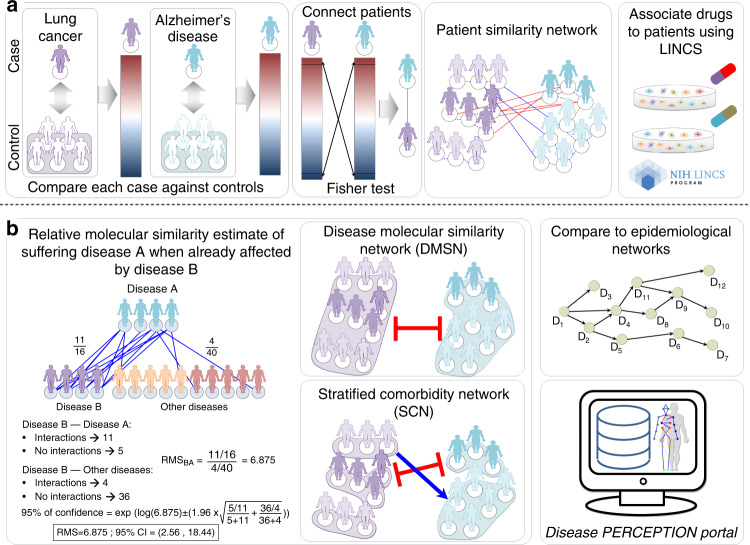
Summary of the steps followed for the comorbidity analyses conducted using transcriptomic data. **a** Generate differential gene expression profiles for each patient (comparing their expression with the expression detected in the control samples), selecting the top 500 up- and downregulated genes for the Fisher’s exact test analysis. Then, use the similarity of these profiles to generate the patient similarity network. Drugs are then associated to patients based on the similarities between patients’ differential expression profiles and the alterations of gene expression generated by a large collection of drugs on cell lines as recorded in the LINCS database (see “Methods”). **b** The number of observed patient−patient interactions connecting different diseases is used to calculate relative molecular similarities as shown, leading to the construction of the Disease Molecular Similarity Network (DMSN) and of the Stratified Comorbidity Networks (SCN). The obtained DMSN is compared to published epidemiological networks and all results are available in the Disease PERCEPTION portal.

To establish patient similarity networks, we collected gene expression data from microarray assays for 6284 patients (and 3887 controls) suffering from 132 diseases and 3 lifestyle conditions (smoking, aging, and exercise) with relative controls. It is worth mentioning that each of the used freely available dataset was conceived for the analysis of a single disease, and so, we cannot know if the patients are suffering from additional diseases. We identified differentially expressed genes (DEGs) by comparing each case sample (from now on patient) to all the control samples from the same study (Fig. 2). We then looked for molecular similarities among patients, based on the significant overlap between the top 500 up- and downregulated genes in each of them^23^ (the procedure was repeated selecting different numbers of DEGs showing this to be the optimal choice; see “Methods”). Studying the molecular similarities between patients based on the expression changes observed in them compared to the controls from the same tissue reduces the tissue of origin effect, as described in our previous study detailing comorbidity relations between Alzheimer’s disease (AD) and non-small-cell lung cancer (NSCLC)^24^. Patients with a significant number of genes deregulated in the same direction (both up- and downregulated, FDR < 0.0001, Fisher’s exact test) were connected by a positive interaction, whereas patients showing overlaps between genes deregulated in opposite directions (upregulated in one patient and downregulated in the other one, and vice versa) were assigned a negative interaction, generating a patient similarity network.

**Fig. 3:**
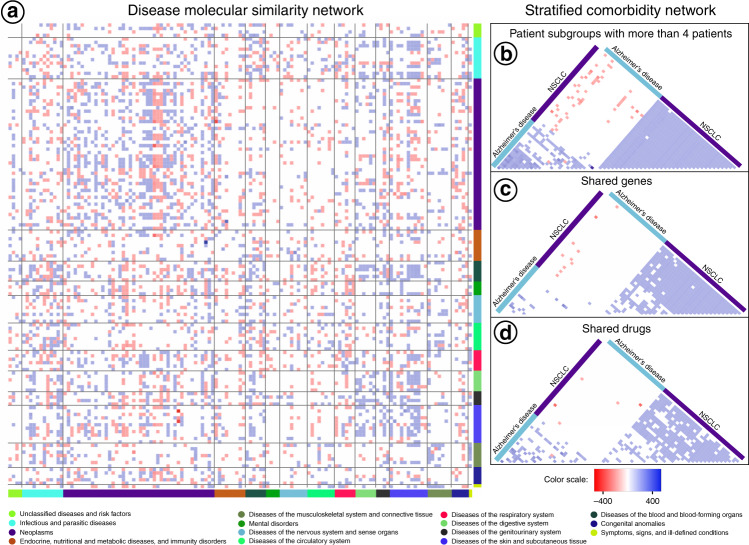
Heatmap representation of the Disease Molecular Similarity Network and the Stratified Comorbidity Network. Blue and red squares represent positive and negative Relative Molecular Similarities (pRMS and nRMS) respectively. **a** Heatmap representation of the Disease Molecular Similarity Network (DMSN). Intensity of the interactions denotes the relative molecular similarity values, interpreted as relative risk interactions, which go from rows to columns. Diseases are colored based on the disease category they belong to (International Code of Diseases, ICD-9-CM). **b** Heatmap of the interactions between NSCLC and Alzheimer’s disease patient-subgroups with at least four patients. **c** Heatmap of the interactions between NSCLC and Alzheimer’s disease patient-subgroups with at least four patients and at least one gene differentially expressed in the same direction in all the patients within the same subgroup. Blue and red squares represent respectively pRMS and nRMS with shared genes in the correct direction (at least one gene differentially expressed in the same direction in all the patients within the two subgroups in the case of positive interactions, and in opposite directions in the case of negative interactions). **d** The same as (**c**) but with drugs instead of genes.

We then generated a Disease Molecular Similarity Network (DMSN) connecting disease-pairs based on the similarities between patients composing them, compared to the similarities with all the other patients (see “Methods”, Fig. 2). We calculated positive and negative Relative Molecular Similarities (pRMS and nRMS) between diseases, hypothesizing that we could interpret them as positive and negative relative risk relations between diseases, using the same approach used in epidemiological studies to calculate relative risks (see “Methods”): a pRMS between diseases A and B could suggest that patients with disease A are potentially at a higher risk of developing disease B compared to all the other patients, while an nRMS interaction could suggest that patients with disease A are at a potentially significantly lower risk of developing disease B than the rest of the patients under study. The resulting DMSN is composed of 135 nodes (all diseases and lifestyle conditions considered in this study), and 4750 edges (Fig. 3a). Most of the interactions were pRMS (56%), which could be interpreted as a potential evidence of direct comorbidity, of which 24% involved diseases from the same International Code of Diseases (ICD9) disease category. Both results were expected, as the presence of the first disease will deteriorate the general state of health of the patient, more likely leading to increased rather than lowered risks for other diseases. Additionally, diseases from the same disease category might share common disease drivers, resulting in a higher co-occurrence probability. This is observed in our study regarding neoplasms, the most connected category, indicating that most cancers showed higher density of molecular similarity relations compared to any other disease categories, in this case as a consequence of the deregulation of cell-cycle-related processes (the complete network can be visualized in the disease PERCEPTION portal, http://disease-perception.bsc.es/index.html). These results are supported by Zhou et al.^25^, who generated a new classification of diseases from disease phenotypes and molecular profiles. In their analysis, they connected diseases based on molecular information (i.e., a combination of GWAS, OMIM and differential expression evidence together with protein−protein interaction networks), detecting that the top ten disease-pairs with the largest number of shared genes are all neoplasms.

According to our hypothesis, our measure of relative molecular similarity should be a reflection of the general comorbidities in populations detected by epidemiological studies. To test this possibility, we first compared our pRMS with an undirected epidemiological network, the Phenotypic Disease Network, generated by Hidalgo et al.^11^ using the disease history of more than 30 million patients (see “Methods”). This network contains relative risks between diseases identified on medical claims regarding hospitalizations for 1990−1993. As in our DMSN, neoplasms were the most connected category (Supplementary Data 1). Interestingly, our DMSN significantly recovers 16% of the Phenotypic Disease Network interactions (541 interactions, empirical *p* value = 0.0088 estimated by randomization (see “Methods”)), a high percentage if we take into account that comorbidity relations could be driven by a variety of factors other than transcriptomics^26^. Our similarity interactions showed higher overlap with the comorbidities involving diseases of the digestive system, diseases of the genitourinary system and diseases of the skin and subcutaneous tissue categories, as well as comorbidities involving neoplasms (Supplementary Fig. 1). On the other hand, it showed a lower overlap with comorbidity interactions involving the diseases of the blood and blood-forming organs. This may be due to a higher variability in cell composition in blood samples, which presents, among other, seasonal and even diurnal variations^27^. Since the relative molecular similarity calculation takes into account the size of the universe of analyzed diseases, larger datasets would be needed to better test the matching of our molecular similarity interactions with epidemiological comorbidity datasets.

**Fig. 4:**
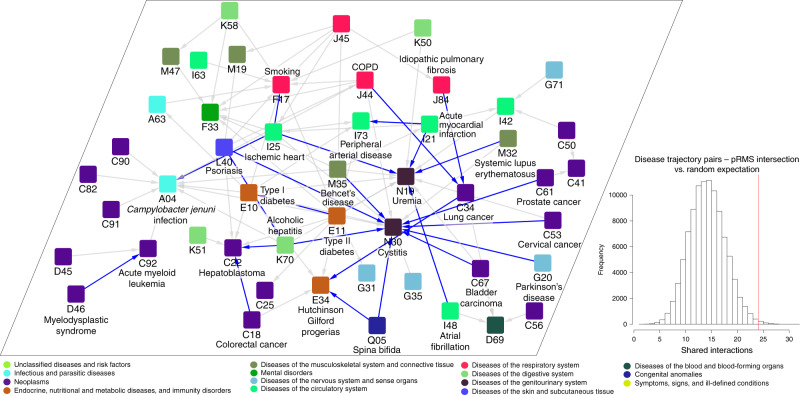
Epidemiologically described disease comorbidities present in the Disease Molecular Similarity Network. Disease-pairs extracted from the Temporal disease trajectories (gray edges) showing which pairs are also detected in the transcriptomic-based comorbidity relations identified by our approach (blue edges). Since the ICD10 codes might involve several diseases, we indicate the specific names of the diseases we are analyzing using transcriptomic data involved in pRMS interactions. Inset: The overlap is statistically higher than what would be expected from randomized datasets (see “Methods”).

We then investigated whether our positive interactions (pRMS) overlapped interactions from a directed disease network, namely the disease-pairs underlying temporal disease trajectories, obtained by Jensen et al.^22^ mining clinical data on 6.2 million patients over 14.9 years. As in the two other networks, neoplasms are still the most connected category. Interestingly, since the temporal disease trajectory network is directed, we can evince that, among our analyzed diseases, the diseases of the genitourinary system are the most common secondary conditions (27% of the disease comorbidity interactions, Supplementary Data 2). Our pRMS pairs overlap 25% of Jensen et al.^22^ disease-pairs (24 interactions, *p* value = 0.0083 estimated by randomization, Fig. 4). Interestingly, 87.5% of the pRMS interactions overlapping Jensen’s network^22^ involve diseases from different ICD categories, suggesting that our measure might reflect more than similarities between diseases. We hypothesize that the overlap between our results and epidemiological studies might be affected, at least partly, by technical issues. Indeed, we have to transform/map disease codes between studies, often losing information. For instance, the very specific disease *Campylobacter jejuni* infection is transformed into very general ICD10 codes (A04, other bacterial intestinal infections). Possibly as a consequence of this transformation, we are not able to detect nine interactions involving A04 code described at an epidemiological level in Jensen et al.^22^ network. When interpreting the significance of this overlap with epidemiological comorbidities, we must consider that gene expression is just one source of information that can be used to reconstruct the comorbidity map. Indeed, compared to other papers that have generated disease−disease interaction networks based on expression data (Hu and Agarwal^7^ and Suthram et al.^28^), we obtain a higher percentage overlap with comorbidity interactions (25% vs. 19% and 10% respectively in the other two studies).

To check whether the number of epidemiological comorbidity interactions overlapping our transcriptomic similarity-based ones can be improved by using other omics data, we downloaded disease−disease interaction networks based on microbiome and miRNA information (see “Methods”). The microbiome-based disease network^10^ recovered six interactions involving our diseases of interest, four of them not detected by our approach (E11-J45, A04-E10, A04-E11, and A04-J44) (Supplementary Fig. 2). In the case of the miRNA-based network^9^, 16 of their interactions overlapped epidemiological comorbidities (not significant, Supplementary Table 1), 9 of them being newly detected interactions. Interestingly, diabetes (either type 1 or 2) was involved in five of those newly detected interactions. The low percentage of overlapping interactions involving diabetes (E10 and E11) and *Campylobacter jejuni* infection (A04) can be a consequence, among others, of the presence of subgroups in the diabetes or mapping problems in the A04 category (regarding the specific global term association mentioned before).

In addition to the pRMS, we also obtain nRMS interactions between diseases, which can be considered as potential evidence of inverse comorbidity relations^23^. These negative relations constitute a new layer of knowledge that cannot be extracted from hospital claims, since a low co-occurrence of two diseases can be a consequence of misdiagnosis or a limited observation window^11^. Indeed, only those studies aimed at specifically evaluating the co-occurrence of a pair of diseases, like the one conducted by Musicco et al.^29^ on the incidence of cancer in patients with Alzheimer’s disease, are able to describe inverse comorbidity relationships. Regarding such comorbidity relations, evidence of the previously described inverse comorbidity relation between AD and NSCLC is also found in our expression-based DMSN^29,30^. Overall, these results suggest that the DMSN network derived from expression data could recapitulate previous epidemiological results, at least in part, potentially being useful in the discovery of the molecular bases of direct and inverse comorbidity relations between diseases. The DMSN represents just molecular similarities between diseases, but exploring the genes involved could shed light onto epidemiologically documented comorbidities.

Finally, we evaluated the association between the pRMS and relative risk measures. To this end, we calculated the Pearsonʼs correlation between our pRMS and the relative risks described by Jensen et al.^22^ in the overlapping interactions. Interestingly, we obtained a significantly positive correlation between the two measures (0.492, *p* value = 0.0146, Supplementary Fig. 3), denoting that the pRMS might be a good estimator of the relative risk of co-occurrence between the two diseases.

### Transcriptome-based patient-subgroups

The general tendencies observed at population level in epidemiological studies (and corroborated by our DMSN) do not necessarily indicate that all patients affected with a disease have higher risks of developing a second one. It is rather possible that just a fraction of patients with a given disease will drive the overall population-wide tendencies to acquire a specific secondary disease, as previously observed^22^. Such differential comorbidity risks might be explained by the existence of disease subtypes, as previously described in diseases such as diabetes^31^ and different cancers^32,33^. The patient similarity network obtained for different diseases allows us to measure diseases’ transcriptional heterogeneity (Supplementary Fig. 4), defined as the percentage ratio between the observed and the total number of possible intra-disease interactions. Since the detection of more than 24 genes deregulated in the same or opposite direction in a pair of patients (Fisher’s exact test FDR ≤ 0.0001) determines the presence or absence of a connection in this network (see “Methods”), transcriptomic heterogeneity must relate to sets of commonly deregulated genes. According to this definition, diseases with few intra-disease interactions, i.e. in which patients are less similar to each other, have higher transcriptional heterogeneity. The ICD9 categories: diseases of the skin and subcutaneous tissue, symptoms, signs, and ill-defined conditions (a category which includes septic shock) and neoplasms are the ones with the lowest transcriptional heterogeneity between patients (Supplementary Fig. 4). On the contrary, mental disorders and diseases of the nervous system and sense organs are the most transcriptionally heterogeneous ones (Supplementary Table 2), potentially as a consequence of diagnostic methods. Such results denote that high transcriptomic heterogeneity might drive different comorbidity patterns in patients affected by the same disease. High heterogeneity can potentially reflect the presence of molecular disease subtypes^31^, when patients perfectly fit the subgroups but not the disease label. Interestingly, we obtained a significant correlation between the transcriptomic heterogeneity and the number of associated symptoms (see Supplementary Notes). Alternatively, high heterogeneity can also be a consequence of low specificity in the diagnostic procedures. Indeed, conditions diagnosed using more accurate methods, like biopsies used for neoplasms, show lower transcriptomic heterogeneity compared to others based on neurocognitive evaluation, e.g. central nervous system disorders. Patient-subgroups in diseases can also be due to genetics and/or to the environment^34^, more explicitly living conditions, food and drug intake^26^.

To further analyze the hypothesis on the existence of disease subtypes driving the differential comorbidity risks, we generated patient-subgroups within each disease, based on patients’ differential expression profiles (see “Methods”). In total, 180 patients were left out of any subgroup, while the other patients were classified into 1126 different subgroups, with a mean number of seven subgroups per disease. Subsequently, we retained only those subgroups that presented a significantly higher number of genes deregulated in the same orientation (coordinately deregulated genes, up- or downregulated in all the patients within the subgroup) than expected by chance (randomly shuffling patients and subgroup associations; see “Methods”). This reduced the number of subgroups to 728. A total of 21% of these molecularly homogeneous subgroups included patients coming from different studies (meaning that 36% of the diseases analyzed by multiple studies show patient-subgroups composed by patients coming from different studies). Even large patient-subgroups composed by many tens of patients share genes that are deregulated in the same direction in all the patients, supporting the reliability of transcriptomically defined patient-subgroups. More specifically, the largest patient-subgroup is composed by 105 patients suffering from septic shock, a disease belonging to the symptoms, signs and Ill-defined conditions category. However, this disease is one of the most homogeneous at the transcriptomic level. Among the genes coordinately deregulated in all the patients we detect *TAGLN3* gene, which is a paralog of the *TAGLN2* gene, previously described to influence the activation of T-cell immunity, potentially involved in septic shock^35^.

To quantitatively evaluate the consistency of the patient-subgroups and diseases in terms of patient-similarities, we calculated the intra- and inter-disease/subgroup interaction percentages for each patient (Supplementary Fig. 5). Most patients presented a higher intra-subgroup than intra-disease interaction percentage, which means that they have more interactions with other patients of their subgroup than with patients having the same disease but belonging to another subgroup. Such difference was especially higher in patients affected by more transcriptomically heterogeneous diseases, e.g. mental disorders and diseases of the nervous system, where the inter-disease/subgroup interactions were similar (compared to neoplasms where the inter-subgroup interaction percentage was considerably higher than inter-disease).

In summary, the observed transcriptomic heterogeneity indicates the presence of patient-subgroups, which in some cases might suggest a complementary classification of patients besides traditionally defined diseases. Defining these patient-subgroups from expression data, we provide the conceptual basis to design a clinically relevant patient stratification based on patient-specific comorbidities.

### Generating the Stratified Comorbidity Network

Using the same approach as the one used to construct the DMSN, we next considered patient interactions at the level of subgroups, and generated the Stratified Comorbidity Network (SCN), which has 728 nodes (i.e., the number of patient-subgroups) and 55,664 edges. Exploring disease interactions at this more detailed level could potentially confirm relations observed between diseases, discover new relations not detected at the disease level, and find comorbidities opposite to the ones described at the disease level. Overall, 82% (2468/3024) of the pRMS and nRMS interactions detected in the DMSN between the diseases were corroborated at the subgroup level in the SCN. Additionally, we detected 3949 new interactions not described in the DMSN. Interestingly, among these new sets of disease interactions, 558 (14%) are opposite to the trends observed at the global disease level. This confirms that patients with a specific disease can present different comorbidity relations depending on the subgroup they belong to, as could be expected given the observed disease heterogeneity.

### Molecular similarities between patient-subgroups

To perform a more in-depth analysis of our SCN, we focused on patient-subgroups composed of at least four patients with coordinately deregulated genes (Supplementary Fig. 6 and Supplementary Data 3), selecting only those interactions where genes are detected (see “Methods”; Fig. 3c). The resulting network comprises 272 patient-subgroups and 3552 interactions, which we can translate into 1280 interactions among 87 diseases. Almost half (45%) of these SCN disease−disease interactions, based on the interactions between patient-subgroups, were not present in the DMSN. Comparing the newly detected pRMS interactions (detected in the SCN but not in the DMSN) with Hidalgo’s and Jensen’s epidemiological networks^11,22^, we observed that 42% and 3% of these new interactions, respectively, were described by epidemiological studies. For instance, we observed a higher than expected risk of developing AD in a subset of smokers, a relation observed at the epidemiological level that was also previously suggested in the literature^36^.

Additionally, the selection of the pRMS and nRMS interactions in the SCN based on shared deregulated genes allows a deeper analysis of the molecular bases of the comorbidities described in the DMSN. For example, we detected miR-10a to be involved in the direct comorbidity relation between the Asthma 28 subgroup and NSCLC 17,24,61,73,82,85,95 subgroups. This relation has been previously described by epidemiological studies but was not detected in the DMSN. Supporting the obtained results, previous studies have described that miR-10a controls airway smooth muscle proliferation^37^, which plays a pivotal role in the pathogenesis of asthma, while this miRNA is also deregulated in NSCLC^38^.

**Fig. 5:**
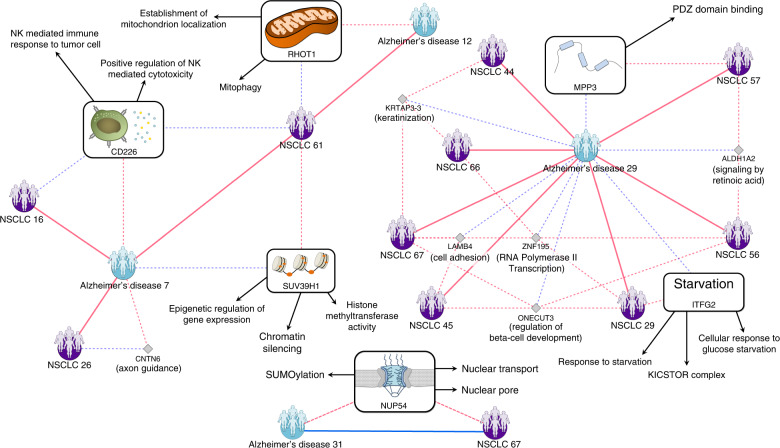
Molecular bases of the Alzheimer’s disease—non-small-cell lung cancer comorbidity relations. Positive and negative Relative Molecular Similarity (pRMS and nRMS) interactions between Alzheimer’s disease and non-small-cell lung cancer subgroups composed by at least four patients with shared genes (extracted from the Stratified Comorbidity Network). Each node represents a patient-subgroup. Red and blue edges represent negative and positive interactions respectively. Solid lines represent interactions between subgroups while dashed lines represent patient-subgroup—gene/pathway interactions. Only those genes (and pathways; see “Methods”) associated to positively or negatively connected patient-subgroups are shown (see “Methods”).

Focusing on the AD−NSCLC relation, we detected 4 out of 25 AD subgroups presenting nRMS relations with NSCLC subgroups. Interestingly, the genes (and their associated pathways) detected to be involved in the nRMS relations of each of the AD subgroups are different, noteworthily associated to immune, starvation, mitochondrial, and methylation processes (Fig. 5). Among others, Natural Killer cells have been separately associated with NSCLC and AD, with a purported proangiogenic role in the former^39^ and decreased cytotoxic functions in the latter^40^. Regarding glucose starvation, lower brain glucose metabolism has been associated with AD^41^, while the activation of glucose absorption and metabolism towards anaerobic pathways characterize the majority of NSCLC^42^.

Interestingly, despite the inverse comorbidity relation (identified in our network as an nRMS) between AD and NSCLC previously described by epidemiological studies and detected also in our DMSN, we detected the AD 31 subgroup to be positively connected (pRMS) to the NSCLC 67 subgroup. Such a result is quite interesting, since despite being inversely comorbid diseases, patients suffering from one can end up developing the second one, as shown by the results obtained for these subgroups. The gene potentially involved in this relation is *NUP54*. Interestingly, *NUP54* associated pathways (Reactome^43^, Kyoto Encyclopedia of Genes and Genome^44^ and Gene Ontology^45^) suggest its involvement in SUMOylation (among others), a process previously described to be associated with both diseases^46^ (Fig. 5). The obtained results highlight the importance of the study of comorbidity at the personalized level, since there can be completely opposite relations between distinct subgroups from the same disease (additional examples are described in the Supplementary Notes, Supplementary Fig. 7).

### Association of differential expression to drug effects

Since gene expression can be altered by drug intake, we investigated if any of the observed interactions could be related to the effects of drugs and small molecules on gene expression patterns, as previously done by Jahchan et al.^47^. To this end, we compared patients’ differential expression profiles with those reported in the LINCS L1000 library (see “Methods”). This database records the gene expression profile changes induced by thousands of compounds on a large panel of cell lines, allowing the user to estimate which compound could generate a given gene expression alteration profile. We added LINCS drugs as nodes in our SCN and investigated whether specific patient-subgroups have any common drug associations. If the changes generated by the drug were similar to the ones observed in specific patient-subgroups, the drug could be responsible for such patterns. On the other hand, if the changes were opposite to the ones observed in patient-subgroups, the drug could serve to treat those patients specifically, opening the way to drug repurposing^47,48^. Our results show that patients within each subgroup had significantly more common drugs associated with them than expected by chance (see “Methods”; Supplementary Fig. 8). Strikingly, using patient−drug associations, we identified different molecular mechanisms potentially involved in the comorbidity between specific patient-subgroups (Supplementary Notes, Fig. 3d), vouching for the importance of a personalized approach to comorbidity relations.

### Patient-specific comorbidity profiles

We have seen that comorbidity relationships can be understood when subdividing diseases into patient-subgroups, suggesting different underlying molecular mechanisms. The generation of patient-specific comorbidity profiles allows the identification of those patients that present significant nRMS and pRMS relations with other diseases. For instance, focusing on the AD−NSCLC inverse comorbidity relation, only 56 and 140 of the AD and NSCLC patients (i.e., 30% and 46% respectively) presented a significant nRMS with the other disease.

**Fig. 6:**
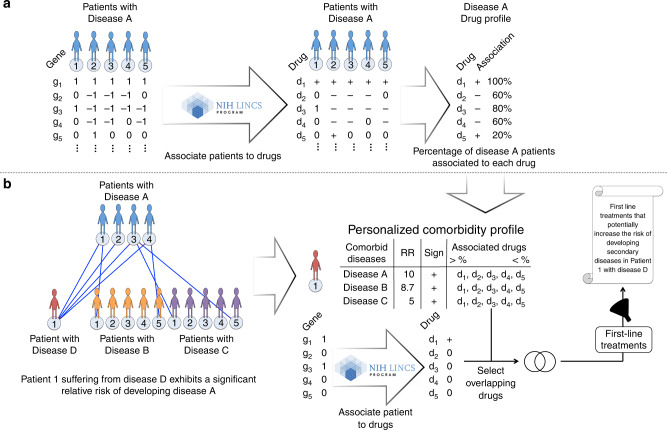
Personalized comorbidity profiles. Two steps are necessary to generate patient-specific comorbidity profiles with associated drugs. **a** Generate a drug profile for each disease. Based on patient-specific differential expression profiles, associate to each patient the drugs that generate expression changes similar or opposite to the ones observed in the patient based on LINCS. Generate a drug profile calculating for each drug within each disease the percentage of patients to whom they are positively associated. Do the same with all the diseases. **b** Generate a personalized comorbidity profile. For each patient, based on molecular similarities, calculate the relative risks of developing secondary diseases based on relative molecular similarities. Associate the patient with the drugs that generate expression changes similar and opposite to the ones observed in the patient using LINCS. Select the overlap between drugs associated with the patient and those associated with each disease. Of those, filter the first-line treatments. The resulting drugs are the ones that might be involved in the increased risk of developing the detected secondary diseases.

The final application of the presented approach is to develop a methodology to predict the most probable comorbidities for each patient. To this end, we considered each patient, evaluated its molecular similarities with all others, and ranked all diseases from the most probable to the least (see “Methods”; Fig. 6), associating LINCS drugs to the comorbidity risks.

We then looked for examples where a patients’ first-line-treatment might be causative of increasing the risk of developing the most probable secondary disease (Fig. 6) based on our transcriptomic data. As an example, we detected one AD patient connected to haloperidol (a traditional antipsychotic drug used to treat psychosis, including the ones suffered by AD patients^49^) with a significant pRMS with interstitial cystitis (interpreted as a significant relative risk of developing the disease). Since 66% of the patients with interstitial cystitis are positively connected to haloperidol, it could be speculated that treating this specific AD patient with haloperidol would increase their risk of developing interstitial cystitis, suggesting that alternative treatments should be sought.

Another remarkable case is the one of cyproterone, an anti-androgen drug usually used to treat hypersexuality in males, severe acne and hirsutism, which has recently been proposed to treat aggressivity in dementia^50^. Interestingly, 23 of the 189 AD patients were negatively connected to cyproterone (pointing to the use of the drug to revert their disease status) and at the same time presented a significant pRMS with astrocytoma (again, interpreted as a significant relative risk of developing the disease). Since 66% of the astrocytoma patients were positively connected to the drug, suggesting that it can cause changes similar to the ones observed in the disease, the drug should be avoided to treat those AD patients’ aggressivity as it might increase their risk of developing astrocytoma.

**Fig. 7:**
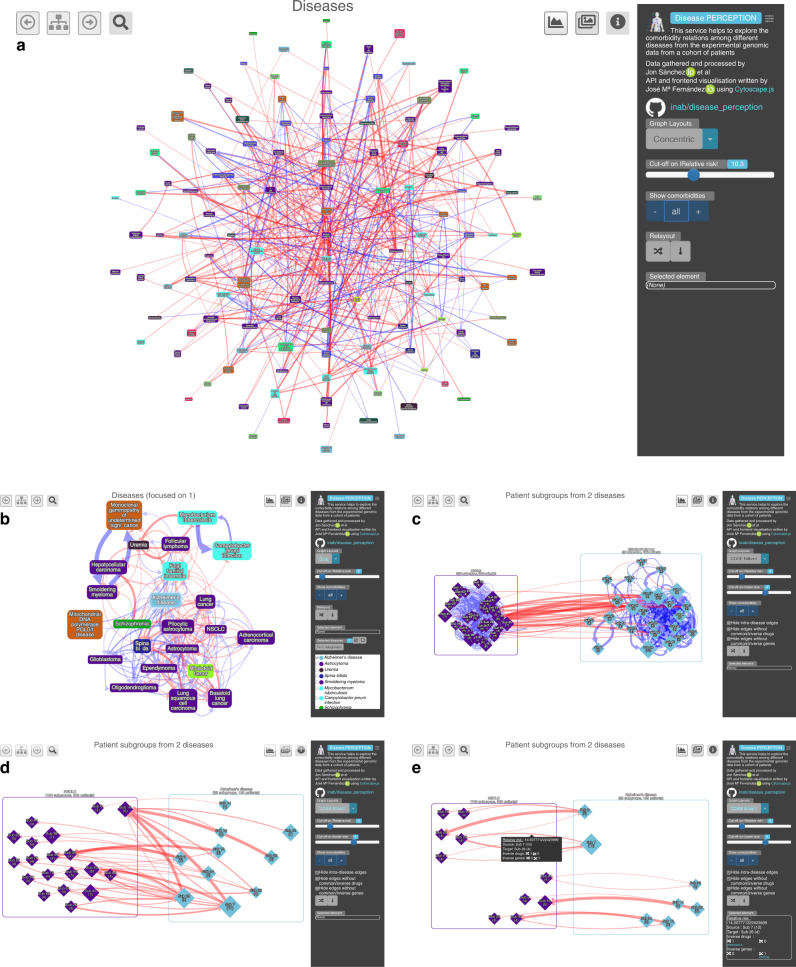
The Disease PERCEPTION portal. Through this user-friendly and programmatically accessible portal, the user can visualize comorbidity relations at the disease and patient-subgroup levels. Moreover, users can extract patient-subgroup information, filtering by subgroup size, intra-subgroup connectivity, as well as by shared drugs and/or genes. Genes and drugs in the networks are hyperlinked to databases, facilitating an interactive exploration of the molecular basis of each connection. **a** Disease network view. Each node represents a disease, colored based on the disease category it belongs to. Blue and red edges denote positive and negative Relative Molecular Similarity (pRMS, nRMS) interactions. Relative risk cut-off can be modified. **b** AD neighbors view. Desired diseases can be selected to show their patient-subgroups. **c** AD and NSCLC patient-subgroups with >4 patients per subgroup. **d** Same as **c** excluding intra-disease interactions. **e** Same as **c** showing only patient-subgroup interactions with shared drugs. Selecting edges of interest displays genes and drugs potentially involved in the selected interactions.

To facilitate the analysis of other cases beyond the proof of principle results reported in this paper, we make all the generated results accessible to the research community through the Disease PERCEPTION portal (Fig. 7; http://disease-perception.bsc.es/), which allows interactive exploration of the Disease Molecular Similarity Network and the Stratified Comorbidity Network.

### Clinical perspectives

Disease subtyping based on gene expression profiles is becoming commonplace in oncology and increasingly in other pathologies like diabetes^31^. Analogously, our results suggest that investigating differential expression profiles could additionally serve to detect the molecular processes potentially driving comorbidities between pairs of diseases, serving in the future for guiding treatment choice. Expression profiles allow the identification of subgroups of patients that might present different physiological states and different comorbidity relations, extending the procedures for disease classification based on the analysis of expression profiles. Indeed, our results show that significant relative molecular similarities can often be related to epidemiologically observed comorbidities, supporting this possibility.

In the future, the richer patients’ molecular phenotypes built with transcriptomics, proteomics and other experimental information will allow a more in-depth study of complex comorbidity patterns and mechanisms, beyond the current picture provided by epidemiological approaches. Indeed, the overlap between the obtained significant relative molecular similarities with the epidemiologically described comorbidities, even when working with different tissues (as in the case of AD−NSCLC relation), suggests that the molecular basis of comorbidities has a systemic character, and profiling patients’ blood samples might be sufficient to produce comorbidity risk profiles, as suggested in other scenarios^51,52^.

It must also be noted that epidemiological detection of comorbidities is far from perfect, since it can be affected by multiple biases, including reporting biases, statistical issues with detection of co-occurrence of rare diseases and uneven coverage of comorbidities spanning different age-ranges. Therefore, we propose the additional use of molecular data to complement epidemiological approaches in the design of management strategies to deal with the important problem for global health that multimorbidities represent^53^.

## Methods

### Gene expression analysis

Gene expression raw data (CEL files) were downloaded from the Gene Expression Omnibus (GEO, GSE* files http://www.ncbi.nlm.nih.gov/geo) and ArrayExpress (EMTAB* files https://www.ebi.ac.uk/arrayexpress/) for 132 diseases, 2 lifestyle conditions (smoking and physical activity) and ageing, including 186 datasets (Supplementary Data 4). Studies conducted on HG U133Plus2 Affymetrix microarray platform were selected to allow using the frozen Robust Multiarray Analysis normalization method^54^ and reduce the bias due to inter-platform differences. The linear regression model provided by the LIMMA package was used to identify differential gene expression^55^, comparing each sample case (from now on denoted as patient) with all the control samples from the same study.

Since the number of significantly DEGs detected varies considerably depending on the disease under study (and thereby directly affects the Fisher’s exact tests), we decided to select a fixed number of up- and downregulated genes. To first evaluate the effect of this selected number of genes for the Fisher’s exact test results, we calculated intra-disease patient-similarities as previously done with diseases^23,24^ using the same threshold for the interactions (Fisher’s exact test, FDR ≤ 0.05) while varying the number of selected genes. As can be observed in Supplementary Fig. 9, the number of detected intra-disease interactions increased with the number of DEGs selected, with a stronger increase in the 100−500 range and then increasing linearly. We therefore decided to continue the analysis using the top 500 up- and downregulated genes based on the *t* values provided by LIMMAs’ differential gene expression analyses.

### Patient similarity network generation

As previously mentioned, we followed a similar strategy to that reported by Ibañez et al.^23^ on disease−disease similarities. Overlaps between pairs of patients were assessed by one-tailed Fisher’s exact tests on lists of DEGs. Two patients are positively connected if they present significant overlaps between genes deregulated in the same direction (both up- and downregulated). On the other hand, two patients are negatively connected if they present significant overlaps between genes upregulated in one patient and downregulated in the other one, and vice versa. If both types of significant overlaps are detected in the comparison (e.g. significant overlaps between genes up- and downregulated in the two patients, and between genes upregulated in one patient and downregulated in the other one), we considered there is no link between those patients. This condition removes 55,086 positive and 24,950 negative potential interactions between patients, that is 2% and 15% of the positive and negative interactions, respectively (Supplementary Fig. 10).

Then, we looked for the one-tailed Fisher’s exact test threshold that did not produce random interactions. To this end, we generated 1000 patients randomly selecting 500 genes as up- and 500 genes as downregulated. We calculated the similarities (both positive and negative) between the generated patients varying the Fisher’s exact test threshold. We repeated this process 100 times and calculated the mean number of patient interactions (independently of the direction of the interaction). As expected, the number of detected interactions in this randomized set decreased while decreasing the threshold (Supplementary Fig. 11). Based on the obtained results, we generated the patient similarity network calculating patient−patient interactions using an FDR threshold of 0.0001. We repeated the analysis varying the number of DEGs selected (100, 200, 300, 400, 1000, 1500, 2000, 2500, 3000, 3500, 4000, 4500, 5000), obtaining for each of them the optimal threshold to avoid detecting random interactions (Supplementary Fig. 12).

### Relative Molecular Similarity estimates

For each pair of diseases, we consider a contingency table (Table 1) counting the number of positive interactions connecting patients from the two diseases and the ones connecting one of the diseases with other ones.

**Table 1:** Contingency table.

	Disease B	No disease B	Total
Disease A	$\frac{{N_{\mathrm{{ab}}}}}{{({\mathrm{{interactions}}}\;{\mathrm{{of}}}\;{\mathrm{{interest}}})}}$	$\frac{{N_{\mathrm{{anb}}}}}{{({\mathrm{{other}}}\;{\mathrm{{interactions}}})}}$	*T*_a_
No disease A	$\frac{{N_{\mathrm{{nab}}}}}{{({\mathrm{{interactions}}}\;{\mathrm{{of}}}\;{\mathrm{{interest}}})}}$	$\frac{{N_{\mathrm{{nanb}}}}}{{({\mathrm{{other}}}\;{\mathrm{{interactions}}})}}$	*T*_na_

We can then define the proportion of interactions connecting patients from the two diseases of interest to the total number of interactions of disease A (1) and the proportion of total number of interactions connecting patients of disease B and diseases other than A, compared to the total number of interactions outside of disease A (2). Positive interactions between patients are considered as interactions of interest, merging both negative and no-interactions as into the “other interactions” category.

1 $$P_{\mathrm{{exposed}}} = \frac{{N_{\mathrm{{ab}}}}}{{T_{\mathrm{{a}}}}},$$

2 $$P_{{\mathrm{{notexposed}}}} = \frac{{N_{\mathrm{{nab}}}}}{{T_{\mathrm{{na}}}}}.$$

These quantities allow us to define positive Relative Molecular Similarities (pRMS) for each pair of diseases, according to formula (3).

3 $${\mathrm{{RMS}}}_{\mathrm{{ab}}} = \frac{{P_{\mathrm{{exposed}}}}}{{P_{{\mathrm{{notexposed}}}}}}.$$

Repeating the same procedure using negative interactions (considering positive interactions as no-interactions), we similarly define negative Relative Molecular Similarities (nRMS).

95% confidence intervals were calculated for diseases, patient-subgroups and patient−disease relations using formula (4).

4 $${\mathrm{{LN}}}\left( {{\mathrm{{RR}}}} \right) \pm 1.96 \times \sqrt {\frac{{\frac{{T_{\mathrm{{a}}} - N_{\mathrm{{ab}}}}}{{N_{\mathrm{{ab}}}}}}}{{T_{\mathrm{{a}}}}} + \frac{{\frac{{T_{\mathrm{{na}}} - N_{\mathrm{{nab}}}}}{{N_{\mathrm{{nab}}}}}}}{{T_{\mathrm{{na}}}}}}.$$

Then, the Stratified Comorbidity Network was filtered selecting only those patient-subgroups composed of at least four patients with coordinately deregulated genes, selecting only those interactions where genes are detected. This means that in the case of pRMS, at least one gene should be deregulated in the same direction in all the patients from the two subgroups, while in the case of nRMS, at least one gene should be upregulated in one subgroup and downregulated in the other subgroup (or vice versa).

### Comparison with epidemiological networks

To validate our results regarding comorbidities, we compared them with the ones obtained at an epidemiological level by Hidalgo et al.^11^ and Jensen et al.^22^. Hidalgo et al.^11^ used ICD9 disease codes to associate patients to diseases generating a disease−disease network called the Phenotypic Disease Network, composed by 995 nodes (ICD9 codes) and 104,434 interactions. We therefore grouped our patients manually using the same disease taxonomy (extracted from http://www.icd9data.com) and calculated relative molecular similarities between ICD9 codes as previously done at the disease level, reducing the confidence interval to 99% as in their analysis. To verify the significance of the overlap between our pRMS and nRMS with the ones detected by Hidalgo et al.^11^, we conducted 100,000 randomizations generating random interactions between the common set of 94 ICD9s. The same was done to compare our network with the disease trajectories, a network composed by 681 nodes (ICD10 codes) and 4014 edges^22^, in this case associating our patients to ICD10 codes. This approach was repeated for each DEG selection (from 100 to 5000). As can be observed in Supplementary Figs. 13 and 14, the number of epidemiologically described interactions overlapping the DMSN increases while increasing the number of selected DEGs. The epidemiological interactions retrieved by the DMSN generated selecting a small number of DEGs are recovered also with the largest DEG selection, denoting a consistency on the overlap with epidemiological interactions (Supplementary Figs. 15 and 16). Since the selection of 500 DEGs is the one that allows us to recover the highest number of interactions for the pRMS, without recovering also a significant number for the nRMS^11,22^ (Supplementary Table 3), in the rest of the manuscript we used the selection of the top 500 DEGs.

### Other omics layers

To compare and complement the overlap obtained with epidemiology using different omic layers, we downloaded microbiome and miRNA-based disease interactions. The microbiome-based disease-interaction network was downloaded from Ma et al.^10^, where diseases are positively/negatively connected if they present similar/opposite changes in the microbiota composition (extracted by text mining from microbiome-analyzing publications). miRNA-based disease interaction network was downloaded from Lu et al.^9^, where two diseases are connected if they share at least one associated miRNA. In this paper, the miRNA-disease association was extracted from ~100 PubMed papers. To make both networks comparable with the expression-based network, we transformed disease names into ICD10 codes using the Unified Medical Language System^56^.

### Patient clustering

Same disease patients were clustered into patient-subgroups based on their discretized differential gene expression information (top 500 up- and downregulated genes were assigned 1 and −1 values respectively, while all the other genes were assigned a 0). The optimal number of clusters within each disease was obtained using the Silhouette method^57^, where *k*-means analyses were conducted using Hartigan and Wongs’ algorithm^58^ varying the number of clusters from 2 to the total number of patients within the disease. The number of clusters with the highest silhouette score was selected as the optimal number of clusters, assigning the patients to their corresponding patient-subgroups using *k*-means and measuring the total number of shared genes (genes commonly deregulated in the same direction in all the patients within each subgroup).

Then, patients from the same disease were assigned randomly to patient-subgroups, measuring the total number of shared genes (this process was repeated 10,000 times). Only those patient-subgroups with significantly more shared genes than expected by chance were selected (*p* value ≤ 0.05 estimated by randomization).

To deepen our analysis of the molecular bases of comorbidity relations between patient-subgroups, we filtered those subgroups with at least four patients, selecting only those interactions with overlapping genes coordinately deregulated in the same direction in both patient-subgroups for positive interactions, and in opposite directions for negative interactions.

### Analysis of the transcriptomic heterogeneity of the diseases

To analyze the transcriptomic heterogeneity of diseases based on patient-similarities, which requires at least 24 genes to be deregulated (minimal number of genes needed in the Fisher’s exact test to obtain a significant overlap with the chosen threshold) in the same or opposite direction (depending on the sign of the interaction), we calculated the intra- and inter- disease/subgroup interaction percentages for each patient. When the intra-subgroup interaction percentages were similar to the intra-disease interaction percentages, we define the disease as transcriptomically homogeneous, whereas in transcriptomically heterogeneous diseases we would observe higher intra-subgroup interaction percentages compared to the intra-disease ones.

### Genes’ associated pathways

To facilitate the molecular interpretation of the comorbidity relations between patient-subgroups, as well as of the biological characteristics of each of them, we looked for the pathways to which each of the detected genes belong in Reactome^43^, Kyoto Encyclopedia of Genes and Genome^44^ and Gene Ontology^45^.

### L1000 LINCS analysis

The LINCS L1000 library is a large catalog of gene expression signatures in cancer cell lines induced by drug treatment or gene knockdown^59^. The *t* values of differential gene expression obtained for each patient when compared against all the control samples in each study were used as gene expression signatures of the patients, and compared against the LINCS L1000 library (http://www.lincscloud.org/), as performed previously^24^.

From the L1000 library, drug-induced expression signatures were obtained from experiments in which the transcriptional state of the cell is measured before and after the treatment with the drug. This allows to study the transcriptional effect of the drug. In order to obtain consensus expression signatures for each drug, a differential expression analysis was performed on control vs. treated cells using Limma^55^.

In the LINCS L1000 data, all the wells in which the same drug was used were considered as treated samples. All the dimethyl sulfoxide-treated wells from all the plates with at least one treated well were considered as untreated controls. The plate in which the drug was tested was taken as a covariate in the expression analysis. As only one type of cell line is used in each plate, using this covariate we take into account the technical batch due to different plates and the biological variability due to different cell lines. Different drug concentrations and exposure time to the drug were not taken into account. In the LINCS L1000 data some drugs (pert_iname) are represented by different molecules, (pert_id) usually from different vendors. In these cases, we obtained the pert_id associated signatures, that is, associated to the molecule, and a consensus signature in which all the pert_id corresponding to the same drug were considered. In this last case, the pert_id was also taken as a confounding variable.

The *t*-moderated statistic was used as a measure of the expression of the gene. It was preferred over the logFC because the *t* statistic takes into account the sampling variance. However, both statistics were highly correlated in all the signatures tested.

In order to measure the similarity of each patient signature to a given drug signature, the enrichment of the top 250 upregulated and downregulated genes by the drug was determined in the patient signature using a pre-ranked GSEA, as previously done by Iorio et al.^48^.

The fgsea R package was used^60^. A consensus Enrichment Score (ES) was obtained subtracting the ES values of the DN signature from those ES of the UP signature.

### Personalized comorbidity profiles

For each patient we calculated the pRMS and nRMS with each of the analyzed diseases based on transcriptomic similarities with other patients, as done before with diseases and patient-subgroups, producing a ranked disease list from the most similar to the least. Then, for each disease we added LINCS drugs and ranked them from the one similar to most patients to the one similar to the least, highlighting the first-line treatments (https://www.vademecum.es). As a final step, we look for examples where a patients’ first-line treatment might be causative of increasing the risk of developing the most probable secondary diseases (Fig. 6), i.e. drugs that are positively connected to most patients of the secondary diseases.

### Disease PERCEPTION portal

The portal is composed of a database loader, an SQL database, a REST API and a web frontend. The tabular data and the source code of the database loader, REST API and web frontend are available at the GitHub project https://github.com/inab/disease_perception.

The database loader is written in Python 3.5, and it uses pandas^61^ and SQLite to prepare an SQLite database instance. The SQL database is composed of 16 tables, with the disease groups, diseases, patient-subgroups, patients, studies, genes, drugs and their relationships. The data loaded comes from all the results consolidated from the analyses previously described.

The REST API is written in Python 3.5, and it uses Flask, Flask-RESTPlus and Flup. It is available at http://disease-perception.bsc.es/api/, and it is documented using OpenAPI.

The Disease PERCEPTION web frontend is written in Javascript ES7/ES2016, and it uses Cytoscape.js^62^, the external layout plugins COLA, COSE-Bilkent, Dagre and Klay, JQuery, Bootstrap, Tippy and Popper. It is built using yarn, babel and webpack, as described in its documentation on the GitHub repository.

### Reporting summary

Further information on research design is available in the Nature Research Reporting Summary linked to this article.

## Supplementary information

Supplementary Information

Description of Additional Supplementary Files

Supplementary Data 1

Supplementary Data 2

Supplementary Data 3

Supplementary Data 4

Reporting Summary

## Data Availability

All data needed to understand and assess the conclusions of this research are available in the main text, supplementary materials and Disease PERCEPTION portal (http://disease-perception.bsc.es). The raw datasets (whose identifiers are provided in Supplementary Data 4) are publicly available and can be downloaded from ArrayExpress (https://www.ebi.ac.uk/arrayexpress/) and GEO (http://www.ncbi.nlm.nih.gov/geo).

## Code availability

Code is available at https://github.com/jonsv89/Disease_PERCEPTION.
