# Supplementary material for: Interpreting molecular similarity between patients as a determinant of disease comorbidity relationships
Source: Nat Commun. 2020 Jun 5;11:2854. doi: 10.1038/s41467-020-16540-x (PMC7275044; doi:10.1038/s41467-020-16540-x)
Supplement: Supplementary file 1 — Supplementary Information [file 41467_2020_16540_MOESM1_ESM.pdf]

Supplementary Information for

Interpreting molecular similarity between patients as a determinant of disease comorbidity relationships

Jon Sánchez-Valle[1], Héctor Tejero[2], José María Fernández[1,3], David Juan[4], Beatriz Urda[1], Salvador Capella-Gutiérrez[1,3], Fátima Al-Shahrour[2], Rafael Tabarés-Seisdedos[5], Anaïs Baudot[6,1,7], Vera Pancaldi[1,8,9*], Alfonso Valencia[1,3,10*]

*Correspondence to: vera.pancaldi@inserm.fr & alfonso.valencia@bsc.es

This PDF file includes:

Supplementary Notes

Supplementary Methods

Supplementary Figs. 1 to 17

Supplementary Tables 1 to 3

Supplementary Notes

1 - Overlap between GWAS data and epidemiology

To further investigate whether considering other omic data, for example genes associated with specific diseases by GWAS, could give us an additional source of molecular disease similarity, we downloaded the disease-gene associations from the GWAS catalog (v1.0.2) [1]. Disease/trait names were transformed into ICD10 codes using the Unified Medical Language System (UMLS)[2]. Only those disease/traits with a single associated three-digits ICD10 code were selected. We connected those diseases (ICD10 codes) sharing at least one associated gene, and compared the resulting network (composed by our diseases of interest) with the epidemiological network generated by Jensen *et al*[3]. Contrary to what was observed for the expression-based molecular similarity network, the overlap with the epidemiological network was not significant (p-value = 0.071, estimated by randomization). Of the 13 GWAS-derived interactions overlapping with the epidemiological network, 8 are not found in our expression-based molecular similarity network, 4 involve E11 (E11-C25, E11-F17, E11-G31 and E11-J45), which is a disease for which coverage in our expression-based network is particularly low (see Supplementary Figure 2). This suggests that our definition of molecular similarity based on gene expression captures disease relationships distinct from the GWAS approach.

2 - Correlation between diseases' transcriptomic heterogeneity and their number of associated symptoms

To test whether transcriptomic heterogeneity is related with phenotypic heterogeneity, we calculated the correlation between them. To this end, we extracted disease - symptom associations from Zhou *et al.* [4], who connect a disease D with a symptom S if they co-occur in at least one PubMed article. We mapped disease MeSH terms into ICD10 codes

using the Unified Medical Language System [5], calculating the number of symptoms associated with each ICD10 code. Then, we calculated Pearson correlations between our transcriptomic heterogeneity (ranging from 0 to 1, from heterogeneous to homogeneous) and the number of symptoms (3-220), obtaining a significant negative Pearson correlation (r = -0.357, p-value = 0.002787). This observation supports our hypothesis that the described transcriptomic heterogeneity might be related to the existence of disease subtypes.

3 - Intra-disease and intra-subgroup interaction percentages

When estimating the intra- and inter-subgroup interaction percentages, almost all patients (93%) presented strong intra-subgroup interactions (more than 50% of their interactions). Comparing this to the results obtained at the disease level (68% of the patients) shows that patients are classified better in their corresponding patient-subgroups than in the classical diseases. Since the ratio between intra- and inter-subgroup interactions is strongly related to disease heterogeneity, we focused on neoplasms, as an example of a molecularly homogeneous disease category, and on mental disorders, as an example of a molecularly heterogeneous group. As can be seen in Supplementary Fig. 4, 64% of the patients within neoplasms present an intra-disease interaction percentage higher than 92%. This percentage increased to 81% when we calculate intra-subgroup interaction percentages. Interestingly, regarding mental disorders and diseases of the nervous system and sense organs, only 1% of the patients presented an intra-disease interaction percentage higher than 90%, this number increasing to 78% when we analyzed intra-subgroup interaction percentages. These results are coherent with the higher heterogeneity of those diseases stemming from the presence of well-defined patient-subgroups.

4 - Filtering the Stratified Comorbidity Network looking for shared genes allows detecting potential biological processes involved in comorbidity relations

As previously done with genes, the Stratified Comorbidity Network can be used to look for drugs potentially associated with comorbidity relations between pairs of patient-subgroups. Focusing on the AD - NSCLC inverse comorbidity relation, we detected 5 negative Relative Molecular Similarities (nRMS) interactions between AD and NSCLC patient-subgroups associated to at least one drug (i.e., at least one drug is positively associated to all the patients from one patient-subgroup and negatively associated to all the patients from the other subgroup). Interestingly, several drugs targeting different molecular mechanisms were detected in those nRMS interactions, with MST312 (telomerase inhibitor), tryptophan (amino acid used in the biosynthesis of proteins), and antimycin A (antibiotic) among others, suggesting that different molecular mechanisms (derived from the patient-drug associations extracted using LINCS) might explain the same comorbidity relationship between diseases. Regarding MST-312, it has been described that telomerase inhibition shows a strong antiproliferative effect on lung cancer [6] and, at the same time, a significantly accelerated rate of telomere shortening has been described in AD patients [7] (pointing to the idea that the telomerase shortening in AD might be driving the protection against NSCLC development). Tryptophan metabolism is altered in AD influencing the balance of pro- and anti-inflammatory cytokines within the Central Nervous System, and has been proposed as a novel druggable target [8]. On the other hand, a higher tryptophan transport and metabolism has been described in tumors with higher proliferation rates [9], and biomarkers of tryptophan metabolism have also been associated with lung cancer risk [10].

In summary, the use of the Stratified Comorbidity Network filtered by shared drugs and small molecules allows the analysis of the specific molecular processes potentially involved in comorbidities. This approach is especially interesting in those cases in which a set of patients present comorbidity relations opposite to the ones observed at the disease-level.

5 - Biological insights into the Relative Molecular Similarity relations between Alzheimer's disease, asthma, diabetes, COPD and schizophrenia

Keeping our focus on Alzheimer's disease, it has been described to be directly comorbid with asthma, COPD, schizophrenia, and diabetes [11,12,13,14] (known to be comorbid between them, forming a size 5 clique). Among the ten comorbidity relations described at an epidemiological level, DMSN only recovers Alzheimer's disease pRMS interactions with both schizophrenia and diabetes. Additionally, three interactions contradict epidemiological tendencies (asthma with COPD and diabetes, and diabetes with schizophrenia).

Going down to the patient-subgroup level (as done in the Alzheimer's disease - NSCLC case), selecting subgroups with at least 4 patients and one gene deregulated in the same orientation in all of the patients composing the subgroup, we still recover interactions between Alzheimer's disease subgroups and diabetes and schizophrenia subgroups. Additionally, we recover pRMS interactions of COPD with schizophrenia, asthma and Alzheimer's disease, and between asthma and diabetes. Interestingly, we still detect nRMS interactions for asthma with COPD and diabetes (opposite to what was expected based on epidemiological studies, that is, a pRMS).

Looking for potential molecular explanations of such relations, we identified the gene ACTL6A, which plays a role in proteolysis, to be up-regulated in the type II diabetes

subgroup 4 and down-regulated in the asthma subgroup 40. These subgroups have a significant nRMS. Protein degradation has been described to be increased both in insulin-deficient and insulin-resistant humans [15]. At the same time, a differential proteolytic activity has been described between eosinophilic and neutrophilic asthma [16], which can potentially explain this unexpected RMS. On the other hand, asthma subgroup 32 and type II diabetes 4 present a pRMS, sharing the down-regulated gene MYLPF, which has been previously described to be associated both to asthma [17] and diabetes [18].

Finally, schizophrenia is known to be directly comorbid with COPD and Alzheimer's disease.

We detect a pRMS between schizophrenia subgroup 28 and COPD subgroup 39. Exploring the molecular drivers of such pRMS, the gene ADRB3 (an adrenergic receptor) is down-regulated in both of them. Interestingly, polymorphisms affecting adrenergic receptors have been associated with COPD in adults [19], and at the same time, several papers provide indirect evidence that adrenergic receptors may play an important role in schizophrenia [20].

Moreover, we detect a pRMS between schizophrenia subgroup 14 and Alzheimer's disease subgroup 7, sharing the down-regulated gene CNTN6. Supporting our results, deletions of the entire CNTN6 gene have been previously described in neuropsychiatric disorders (including schizophrenia) [21], and at the same time, duplications at 3p26.3 disrupting the CNTN6 gene have been described in Alzheimer's disease [22].

6 - Correlation based similarities

As an alternative approach, patients were also connected based on correlation analyses. In summary, we calculated pairwise Pearson correlations on the complete list of DEGs (t-values provided by LIMMA), selecting only patient pairs with significant positive or

negative correlations (after correcting for multiple testing). Then, we calculated both positive and negative Relative Molecular Similarities (pRMS and nRMS) between diseases, looking for the overlap with epidemiological networks [23,3]. By means of this approach we did not recover significant overlaps between our pRMS and nRMS with Jensen et al. network [3] (Supplementary Table Ans.1, Supplementary Fig. 15), meaning that transcriptomic similarities estimated by correlation of expression values cannot significantly recapitulate epidemiologically described interactions. On the other hand, we obtained significant overlaps between both our pRMS and nRMS with Hidalgo et al. network [23] (Supplementary Data 4, Supplementary Fig. 16), meaning that diseases described to be comorbid on this epidemiological network can present both positive and negative transcriptomic similarities as estimated by DEGs correlation. The obtained results show that correlation-based analyses are not the best option for the purpose of the study.

7 - Patient classification

We have seen that comorbidity relationships can be understood when subdividing diseases into patient-subgroups, suggesting different underlying molecular mechanisms. Therefore, we set to predict the comorbidity propensity of new patients by locating them in our networks, in a proof-of-principle personalized framework. To this end, we associated each patient to the most similar patient, according to the Euclidean distances between their discretized differential expression profiles (see Supplementary Methods), and then assigned them to a disease and patient-subgroup. Using this approach, 92% of the patients were properly assigned to their corresponding disease. We correctly assigned 183 of the 189 patients with AD, of whom 132 were also correctly classified into their corresponding subgroups, and 293 of the 302 patients with NSCLC, of whom 211 were

also correctly classified into their corresponding subgroups (see leave-one-out procedure details in Supplementary Fig. 17).

In order to test the performance of our method with an external independent dataset not previously used, we downloaded one additional NSCLC expression dataset (see supplementary methods), and tested whether these new patients could be correctly associated with their disease. 68% of the patients were classified into NSCLC, and the remaining 32% were classified into lung cancer (not specifying the type of lung cancer).

To conclude, estimating Euclidean distances between discretized differential expression profiles for each patient, we are able to assign patients to their corresponding diseases and importantly to these newly defined subgroups, suggesting the potential for the construction of a general system for the prediction of patients' specific comorbidity risks.

Supplementary Methods

New patient classification and comorbidity prediction (methodological approach)

Each patient in our dataset was classified into their corresponding disease and patient-subgroup using a leave one out approach, comparing the patients' differential gene expression profile with those of all other patients (up-regulated genes were denoted with 1s, down-regulated ones with -1s and all the other ones with 0s) using Euclidean distances. True positive (TP), true negative (TN), false positive (FP) and false negative (FN) values were calculated for each disease, and for each patient-subgroup within the same disease. Precision, recall and specificity values were calculated and compared to random expectation, shuffling the gene expression values across patients.

Then, one new NSCLC dataset with 25 patients, analyzed using the same microarray platform (HG U133 Plus 2), was downloaded from GEO (GSE27262). Patients were classified into one of our 135 diseases and risk factors based on their differential gene expression profiles.

Supplementary Figures

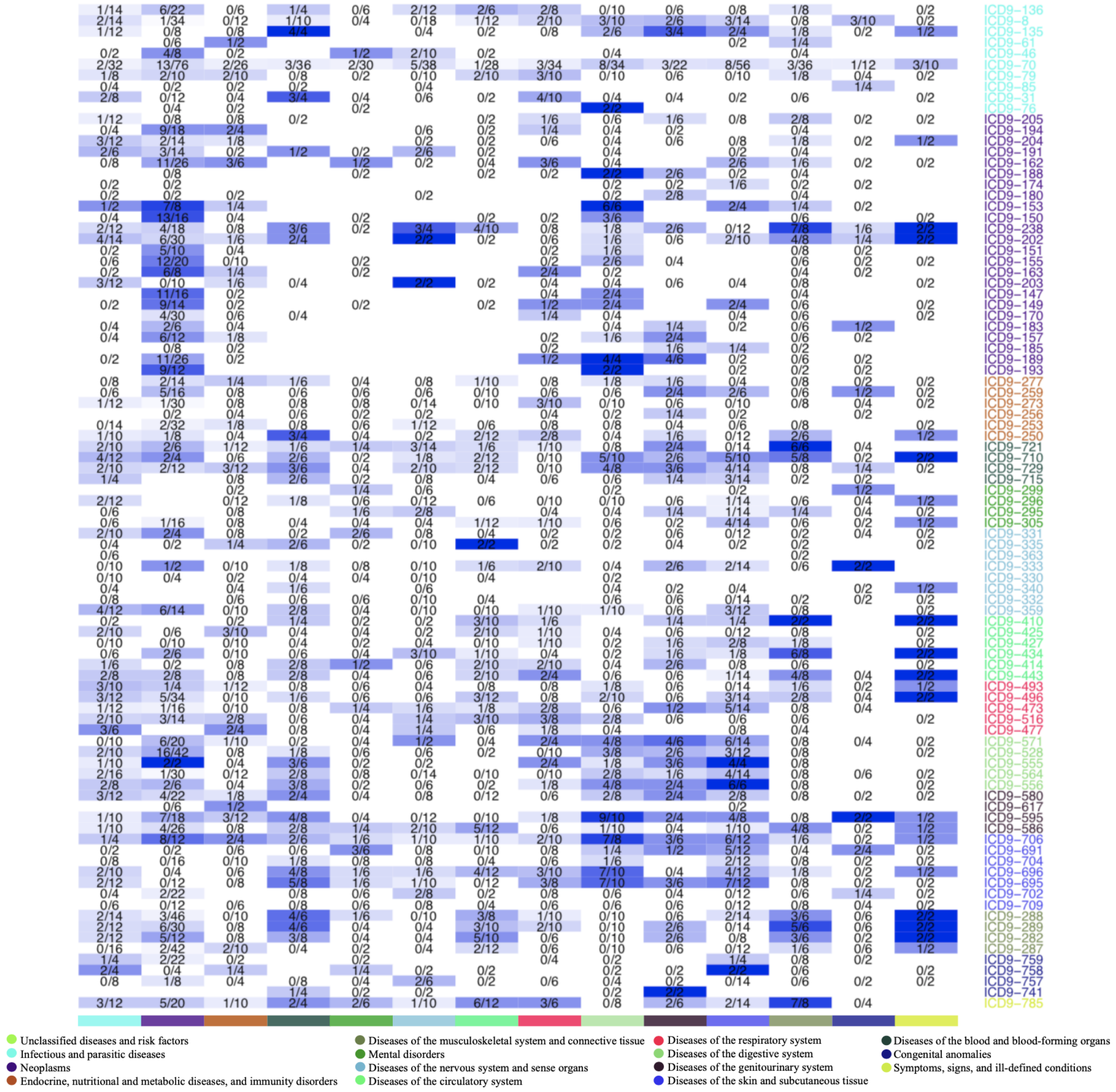

Supplementary Fig. 1. Heatmap representation of the percentage of interactions described by the Phenotypic Disease Network detected by our method. Numbers inside the boxes indicate the number of interactions between the ICD9 codes (on rows) and disease categories (on columns) described epidemiologically that are explained by our approach. The darker the cell, the higher the percentage of interactions explained. Those cells without numbers are interactions that are not described epidemiologically.

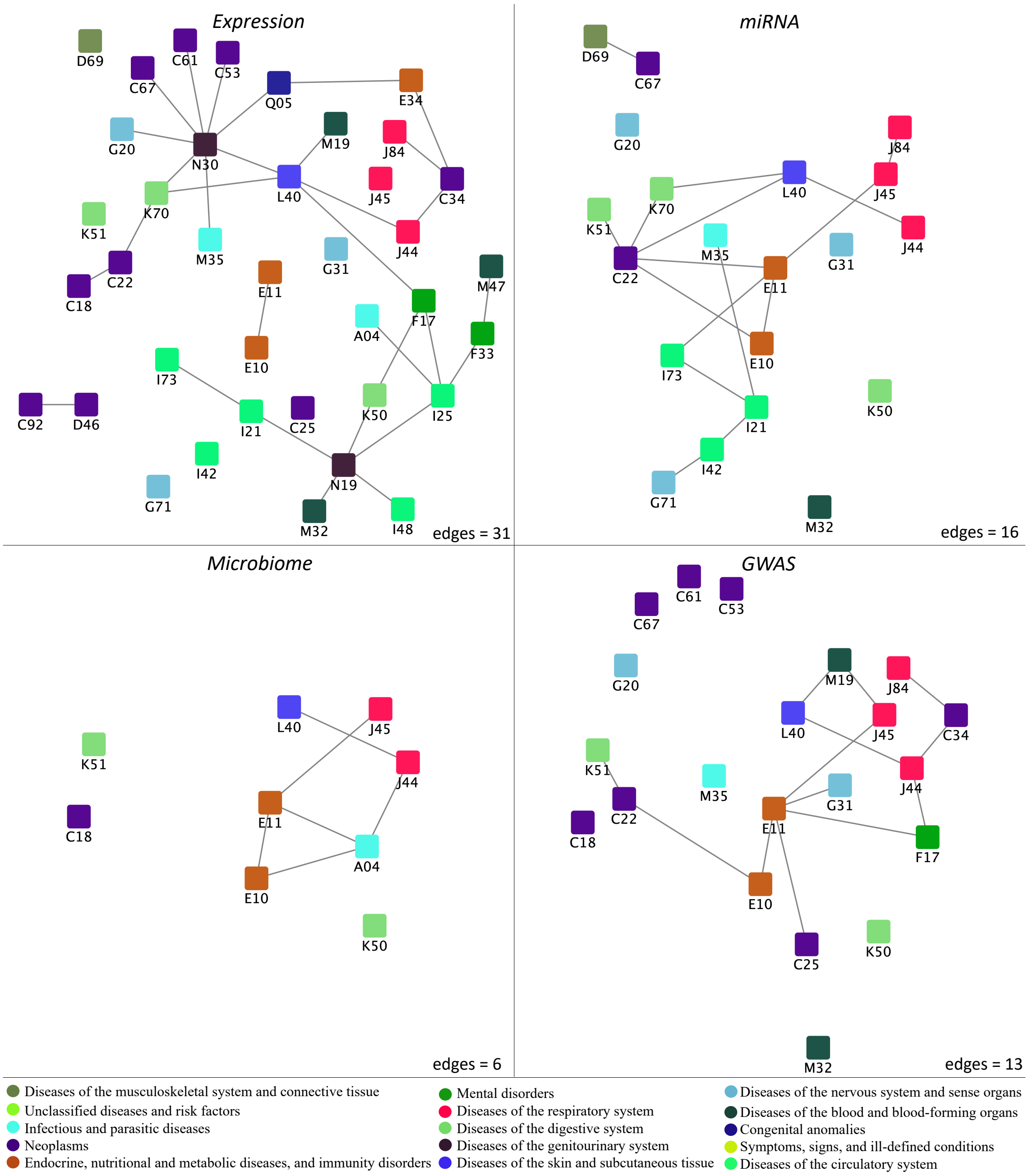

Supplementary Fig. 2. Undirected disease-disease interaction networks extracted from different omics data (expression, miRNA, microbiome and GWAS). Nodes are coloured based on the disease category they belong to. Only the edges identified by epidemiology and the corresponding omic layer are represented. Number of detected edges are denoted in each single layer. Expression (our study), microbiome [24], miRNA [25] and GWAS.

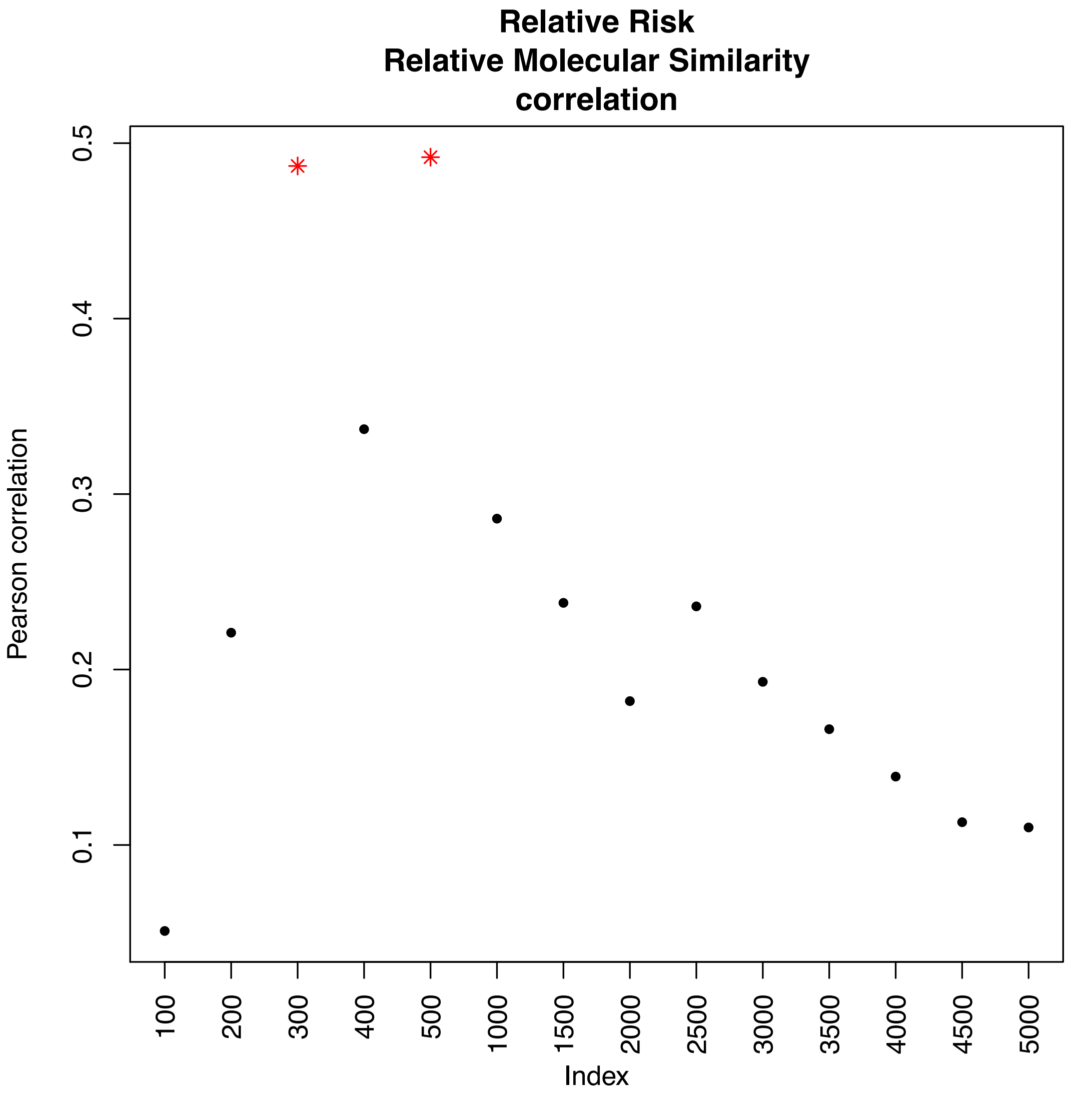

Supplementary Fig. 3. Pearson correlations between positive Relative Molecular Similarity and Relative Risk values selecting different number of differentially expressed genes for the patient-patient similarities. Red asterisks denote significant correlations.

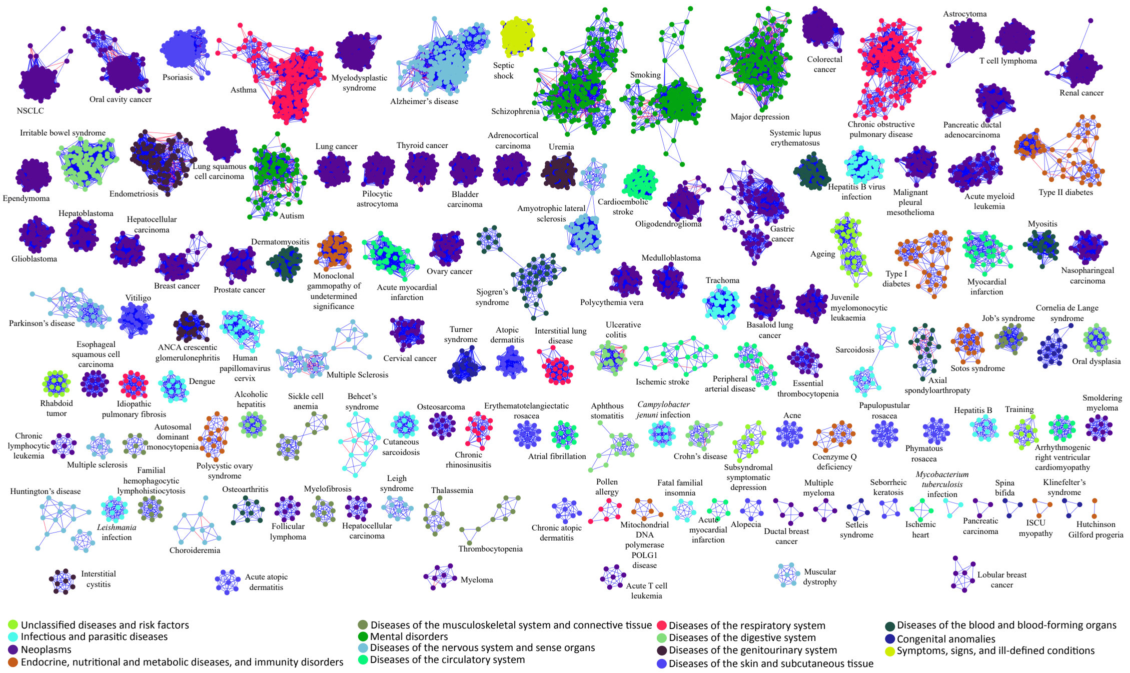

Supplementary Fig. 4. Intra-disease patient similarity network and associated diseases' transcriptomic heterogeneity. Intra-disease patient-patient interaction network. Each node represents a patient. Blue and red edges represent positive and negative interactions respectively. Nodes are coloured based on the disease-group they belong to. Organic layout was used to represent the network using Cytoscape [26].

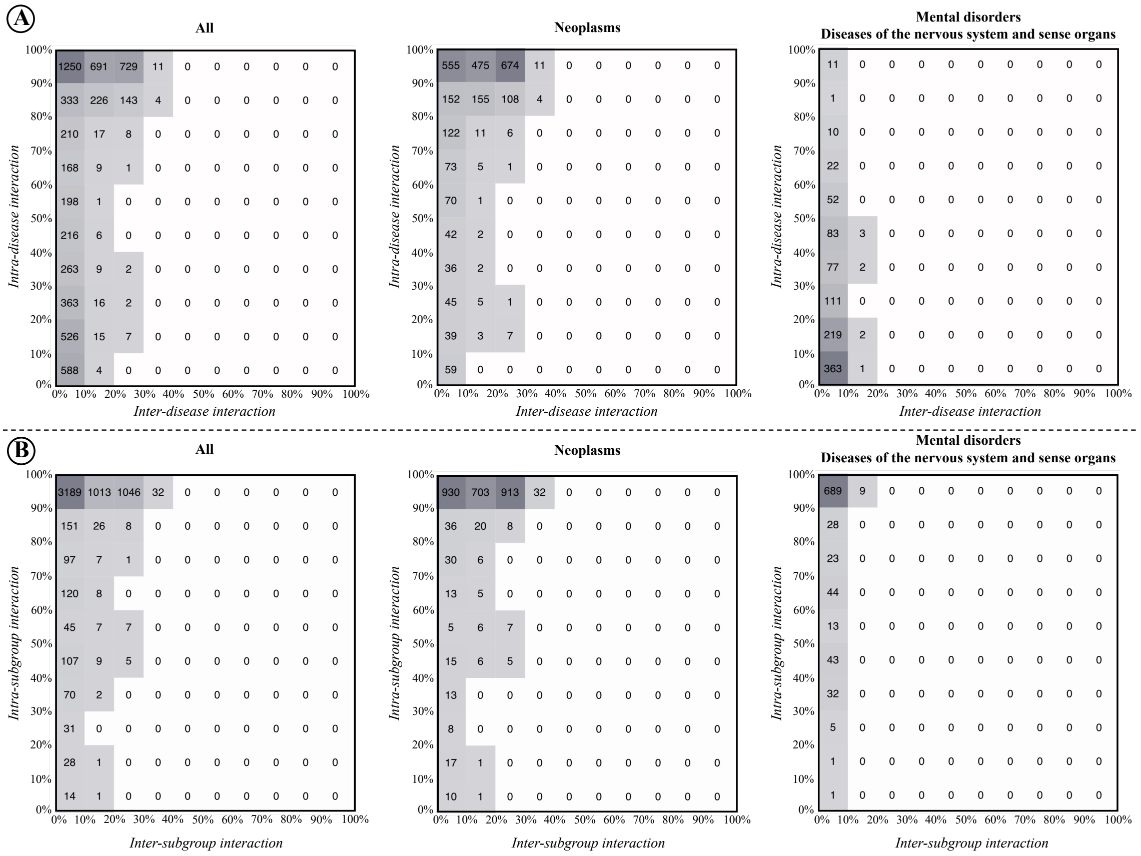

Supplementary Fig. 5. Patients' intra- vs. inter-disease/subgroup interaction percentages. A) Number of patients within each interval of intra- and inter-disease percentage are indicated by the numbers and by the intensity of the colour. **B)** The same as A but referring to intra- and inter-subgroup percentages.

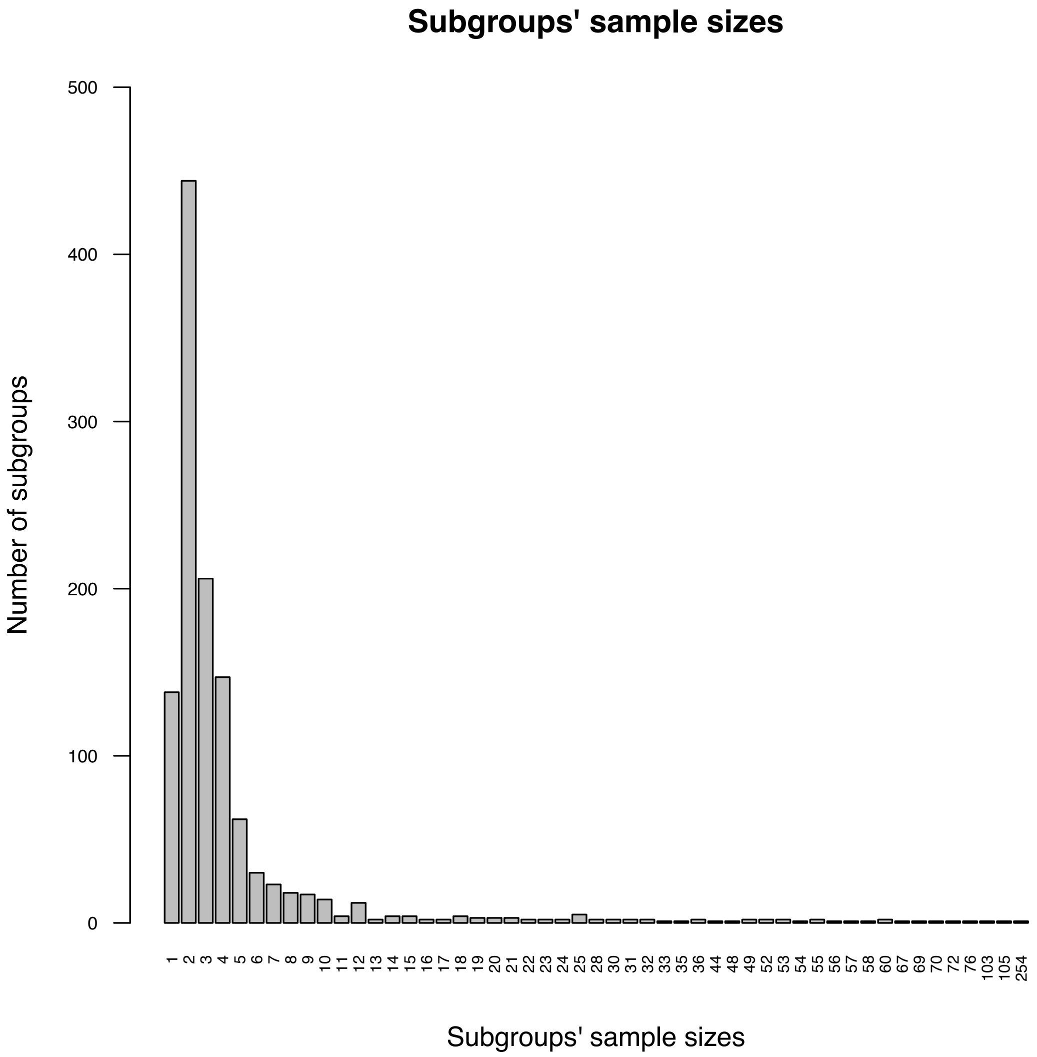

Supplementary Fig. 6. Distribution of patient-subgroups' sample sizes.

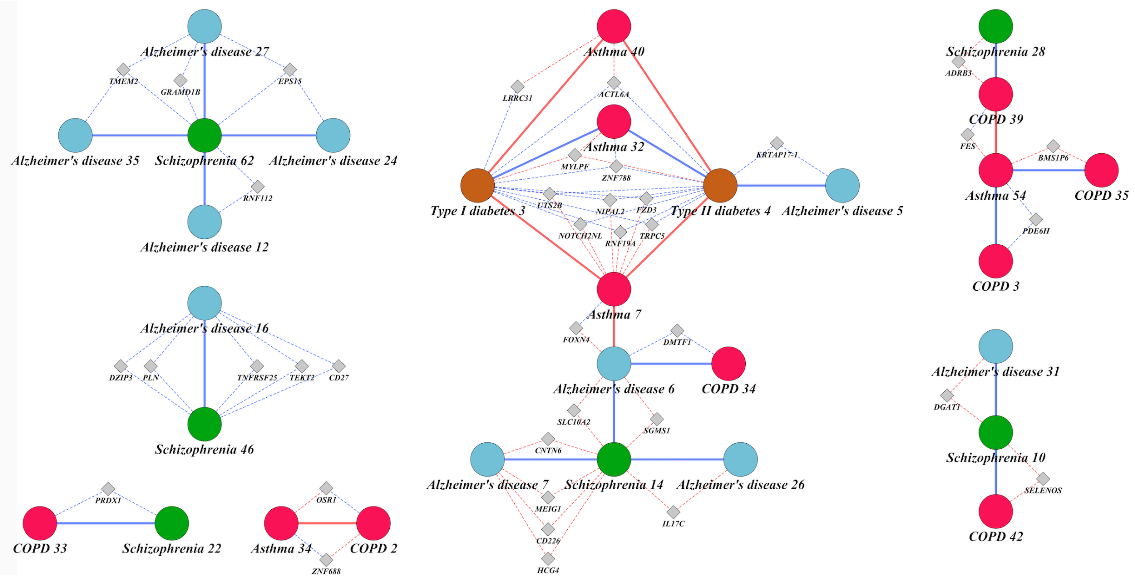

Supplementary Fig. 7. Circles denote patient-subgroups with at least 4 patients. Diamonds denote genes. Solid blue and red lines indicate positive and negative relative molecular similarity interactions respectively (evidence of direct and inverse comorbidities). Dashed blue and red lines indicate that the gene is up- or down-regulated in all the patients composing the subgroup to which is connected. Light blue, green, red and brown nodes represent “Nervous System diseases”, “Mental disorders”, “Respiratory diseases” and “Metabolic diseases” respectively.

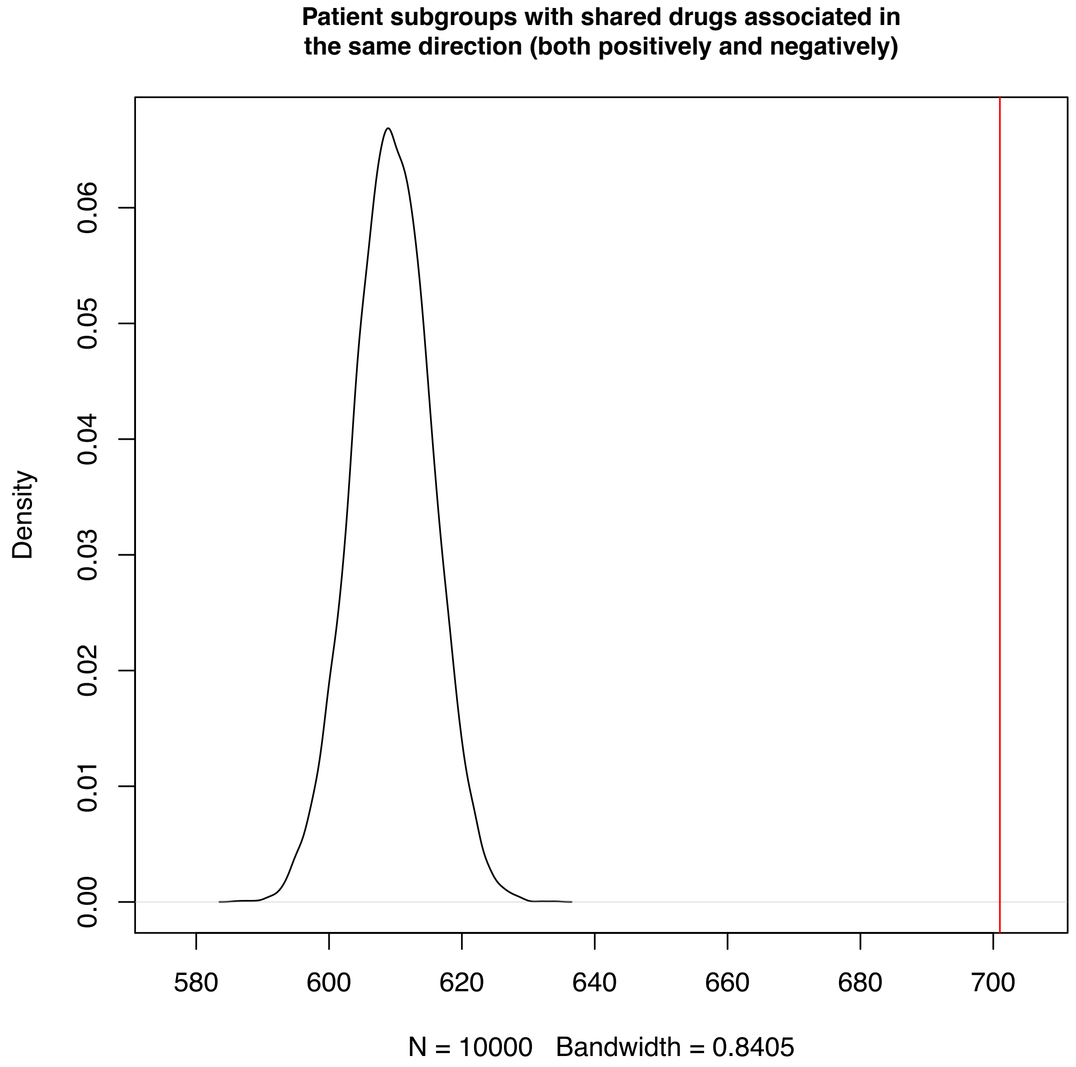

Supplementary Fig. 8. Number of molecularly homogeneous subgroups with shared drugs (red line) against random expectation (shuffling patients with the same disease 10,000 times). Patient subgroups are selected if there is at least one drug positively and one drug negatively connected to all the patients composing the subgroup.

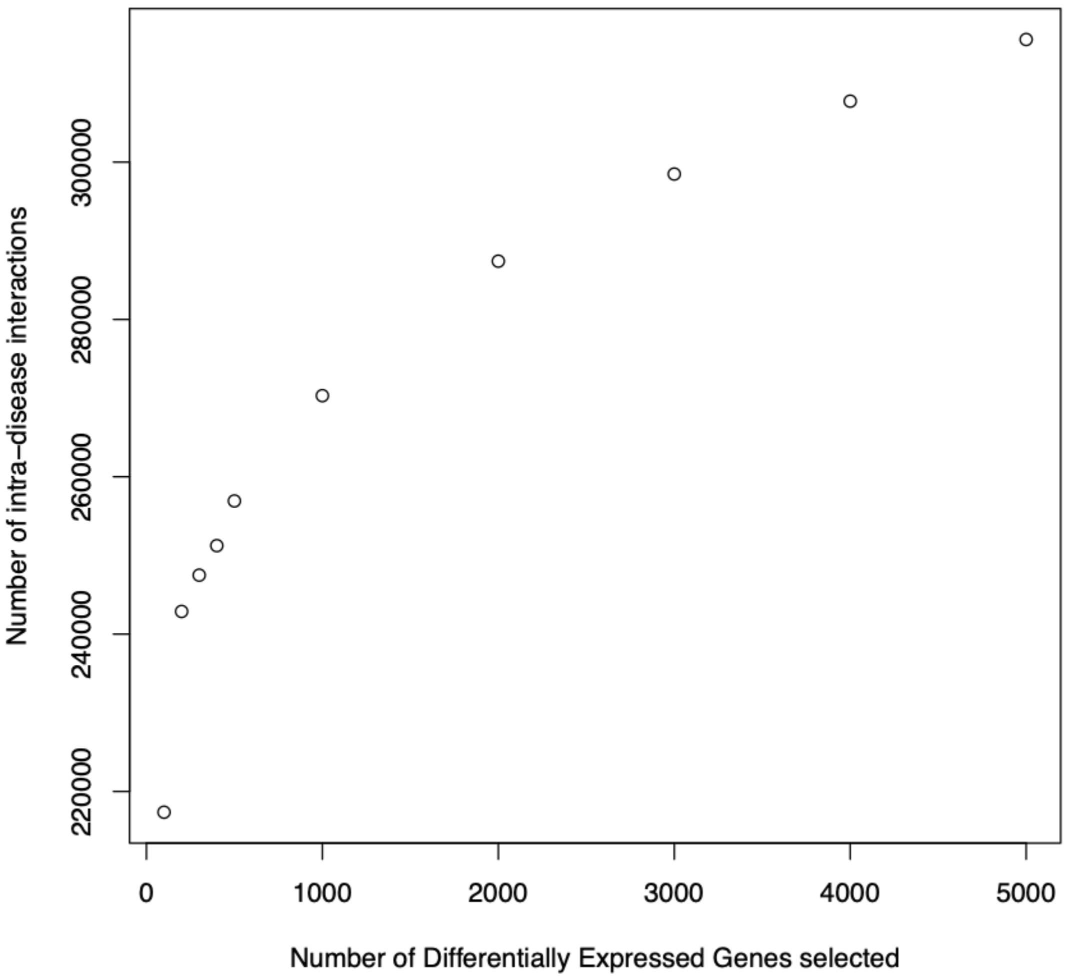

Supplementary Fig. 9. Number of intra-disease interactions detected varying the number of top differentially expressed genes. Number of both positive and negative interactions between patients with the same disease as a function of the number of selected Differentially Expressed Genes.

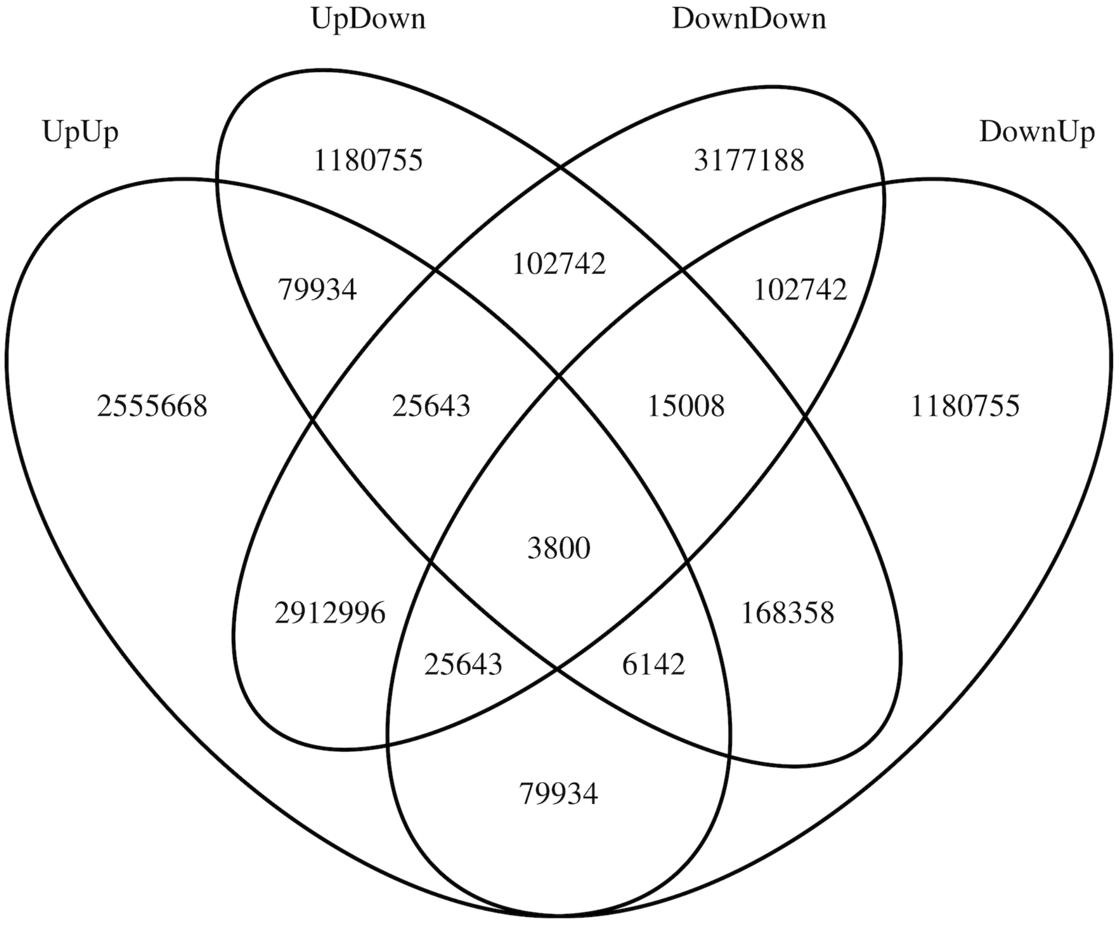

Supplementary Fig. 10. Venn diagram showing the number of significant overlaps (FDR<=0.0001, Fisher's exact test) between the genes deregulated in the same or opposite direction in the pairs of patients. UpUp, UpDown, DownDown and DownUp refer to the cases where there is a significant overlap between the genes up-regulated in both patients, up-regulated in one patient and down-regulated in the other one, down-regulated in both patients and down-regulated in one patient and down-regulated in the other one respectively.

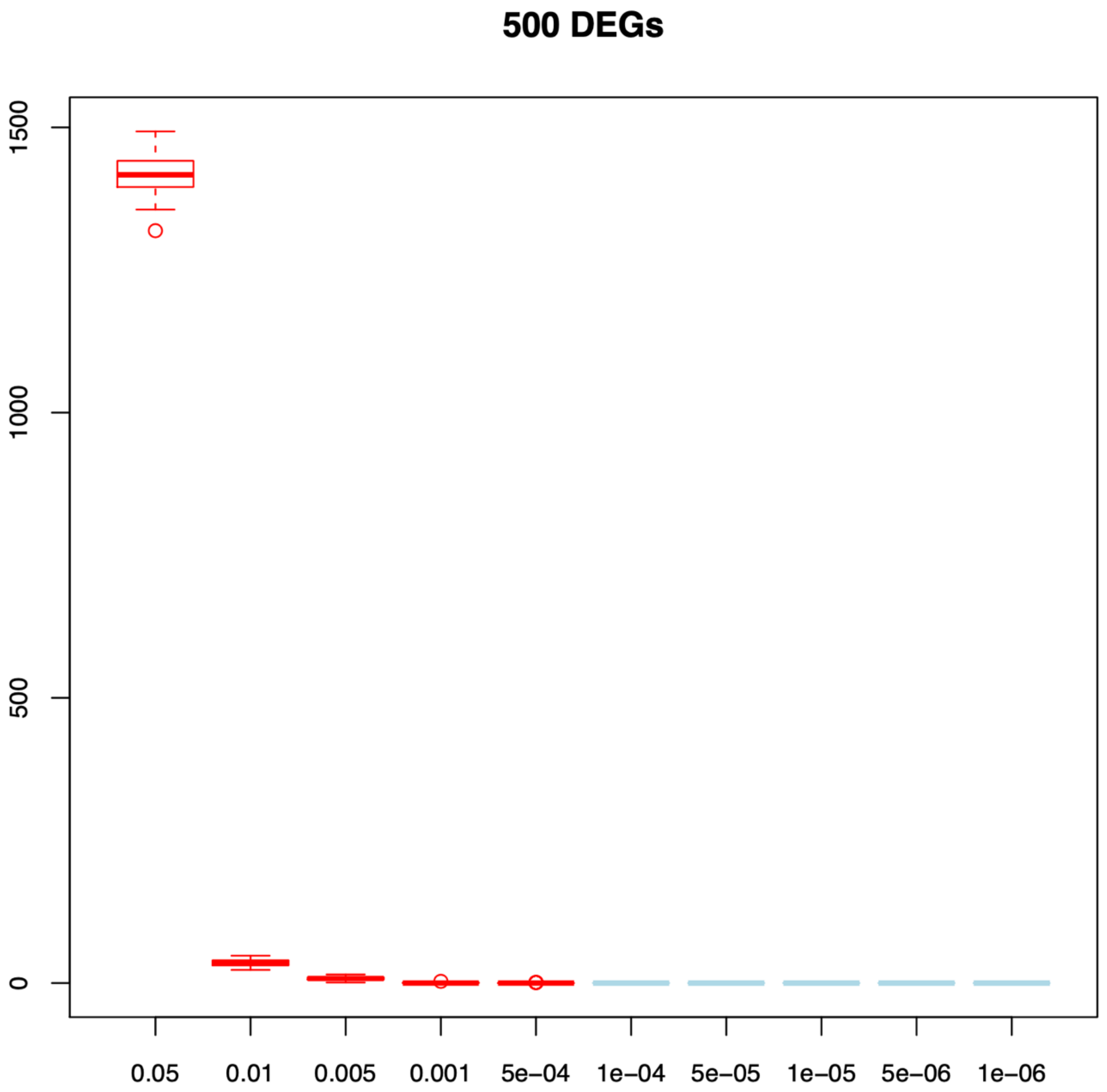

Supplementary Fig. 11. Intra-disease interaction density. Mean number of interactions among 1,000 patients randomly selecting 500 genes as up- and down-regulated after 100 permutations. X-axis represents varying p-value cut-offs on the Fisher's exact test.

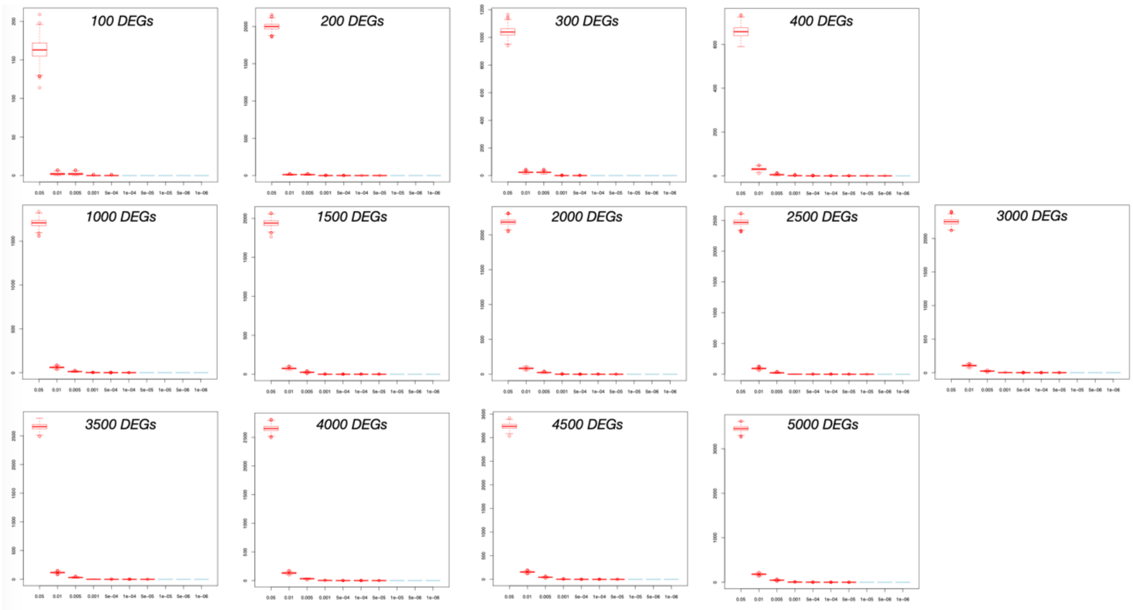

Supplementary Fig. 12. Mean number of interactions among 1000 patients randomly selecting the top 100, 200, 300, 400, 1000, 1500, 2000, 2500, 3000, 3500, 4000, 4500 and 5000 DEGs after 1000 permutations.

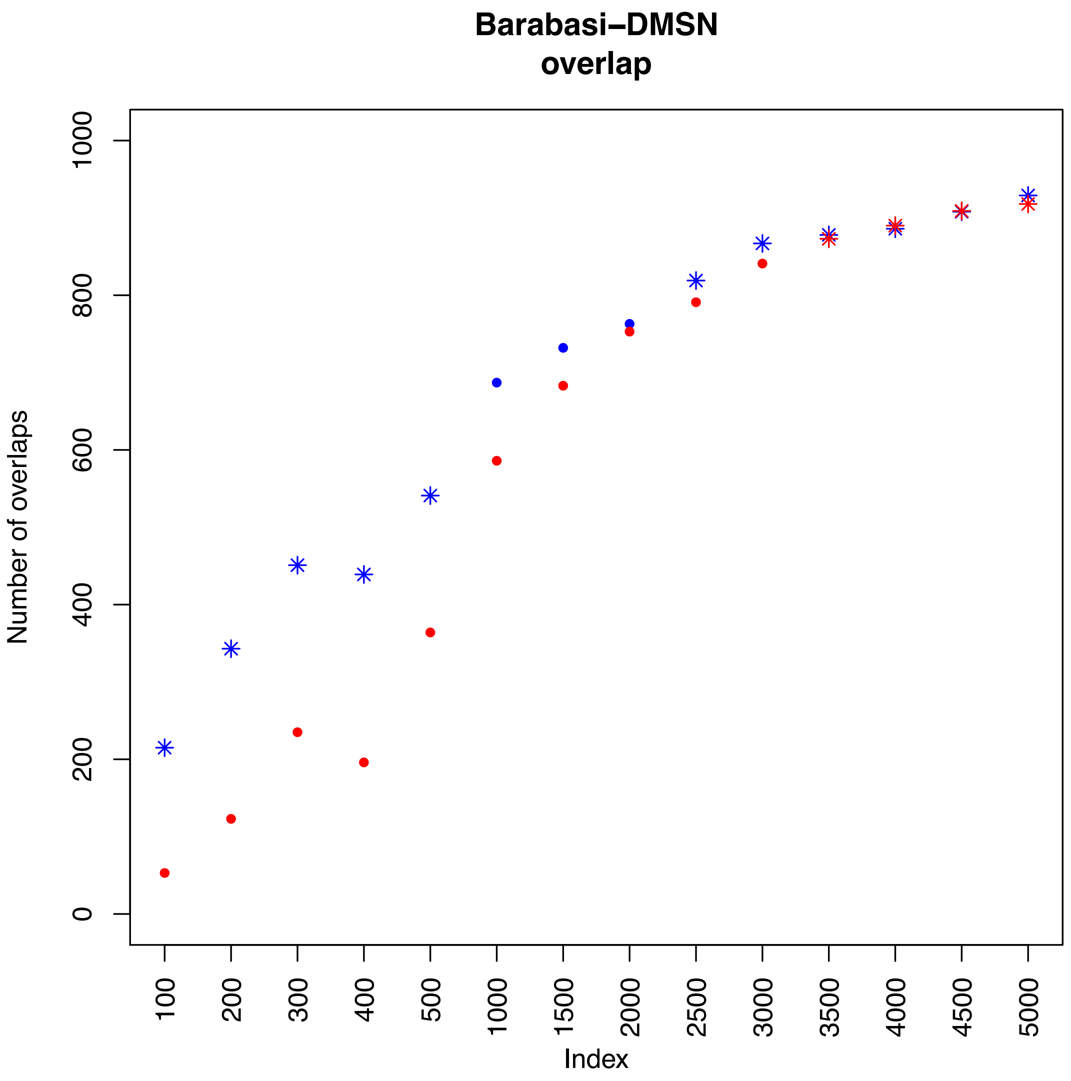

Supplementary Fig. 13. Number of interactions overlapping between Hidalgo´s network [23] (ICD9 codes) and the DMSN generated with each single gene selection. Asterisks and points represent, respectively, significant and non-significant overlaps between both our molecular-based positive (blue) and negative (red) interactions with Hidalgo's network [23].

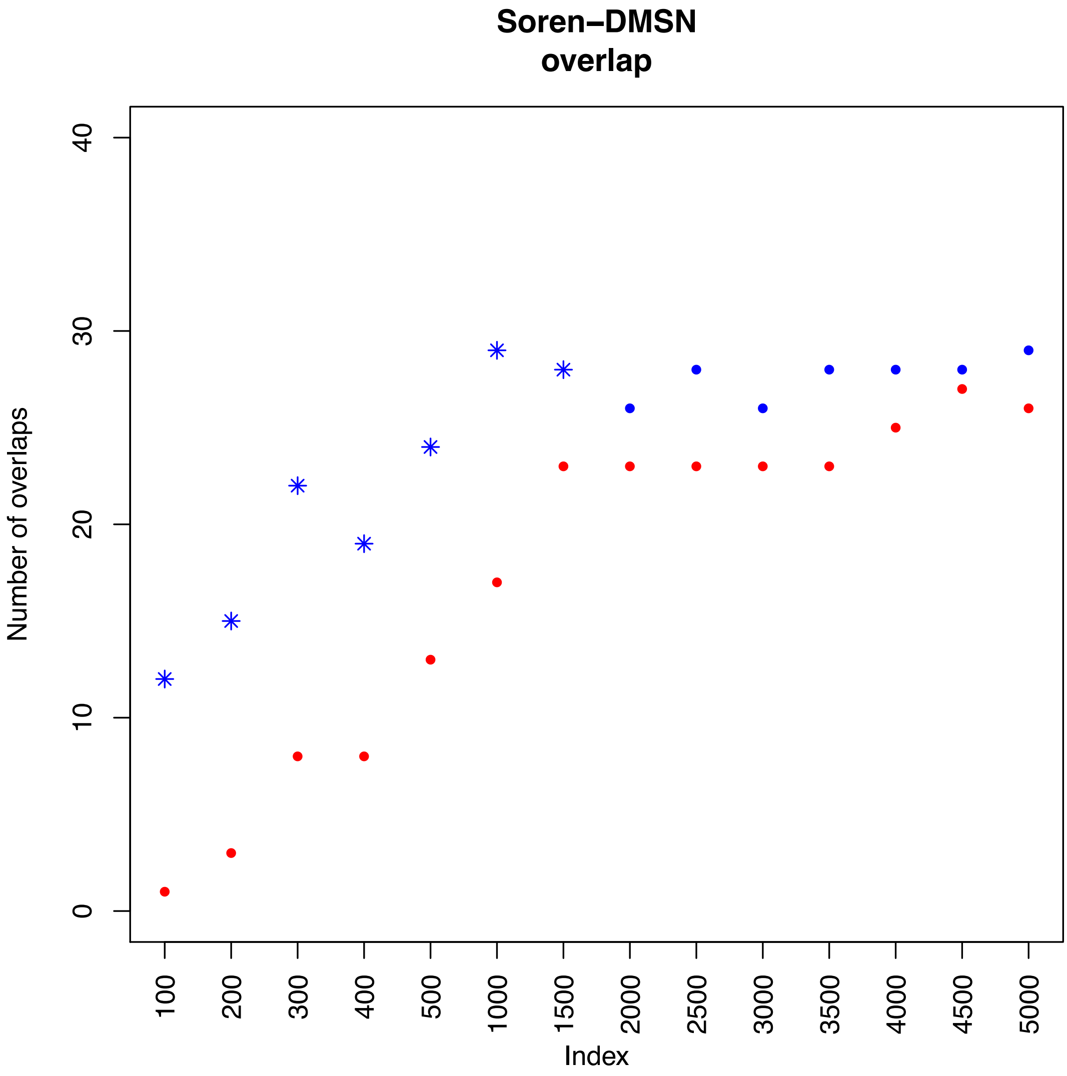

Supplementary Fig. 14. Number of interactions overlapping between Jensen's network [3] (ICD10 codes) and the DMSN generated with each single DEGs selection. Asterisks and points represent respectively, significant and non-significant overlaps between both our molecular-based positive (blue) and negative (red) interactions with Jensen's network [3].

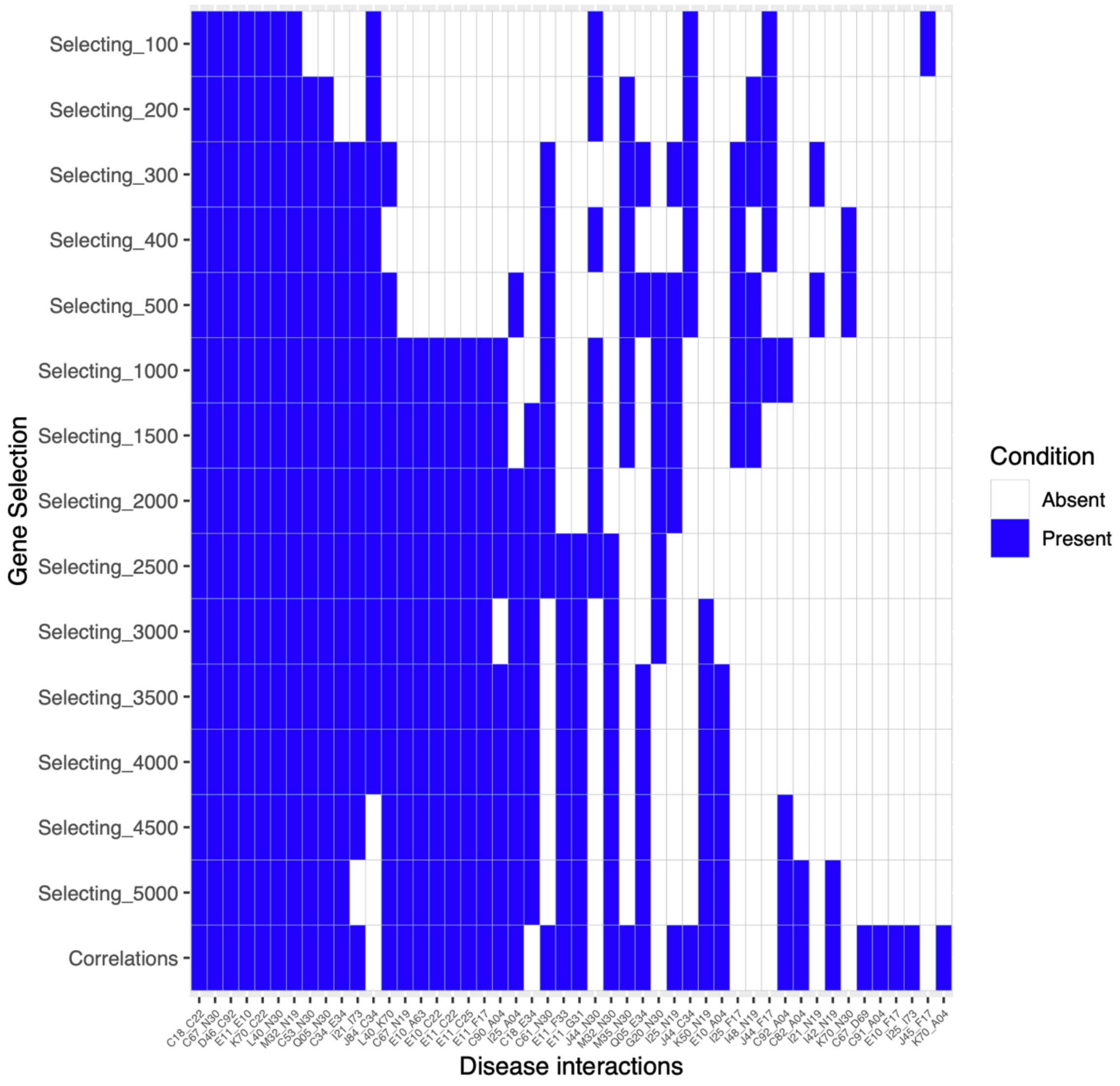

Supplementary Fig. 15. Disease-disease interactions described by any of the DMSN generated selecting different numbers of genes overlapping with Jensen's network [3]. Rows represent the number of selected genes, columns represent disease-disease interactions in ICD10 3 digits codes. Blue squares denote that the interaction has been obtained with the specific selection of genes, while white ones are the ones not obtained with the gene selection.

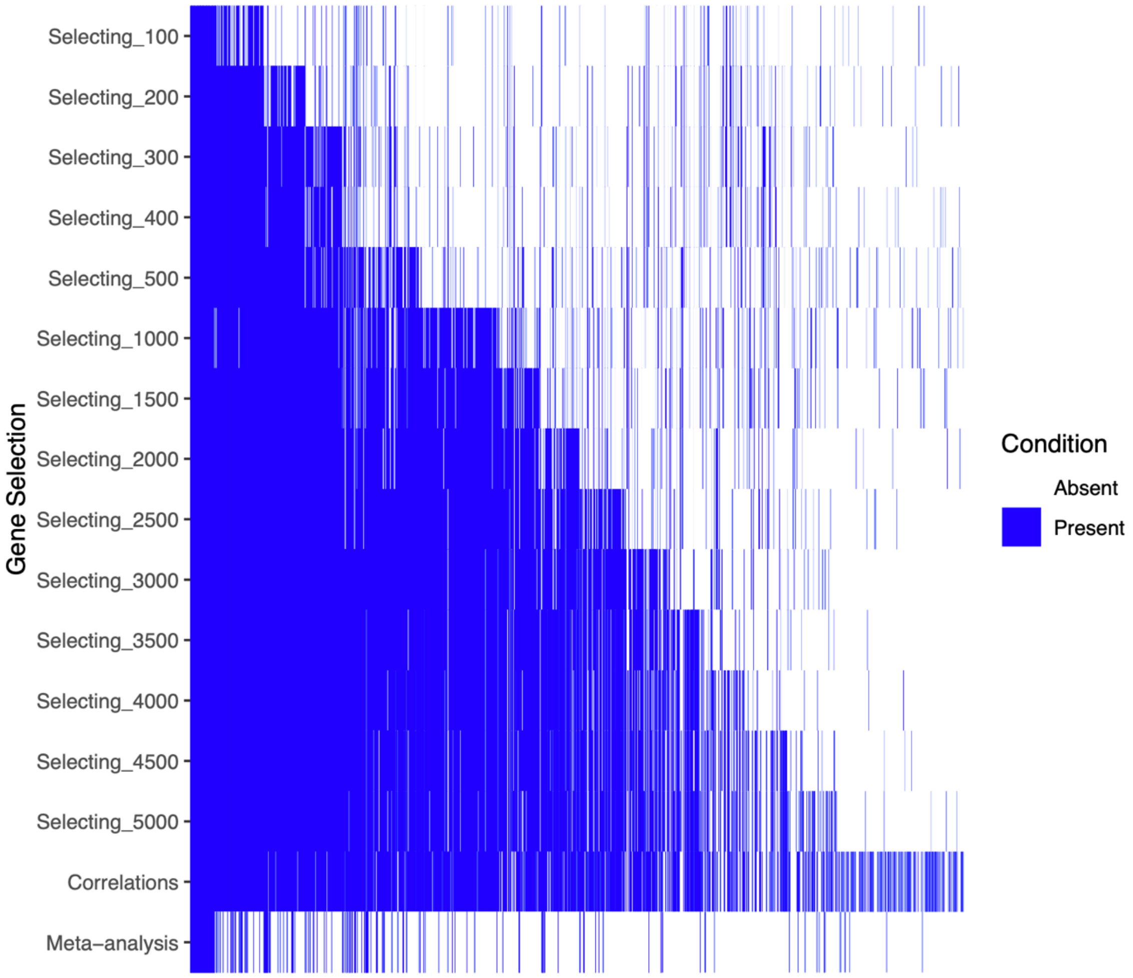

Supplementary Fig. 16. Disease-disease interactions described by any of the DMSN generated selecting different numbers of genes overlapping with Hidalgo's network [23]. Rows represent the number of selected genes, columns represent disease-disease interactions in ICD10 3 digits codes. Blue squares denote that the interaction has been obtained with the specific selection of genes, while white ones are the ones not obtained with the gene selection.

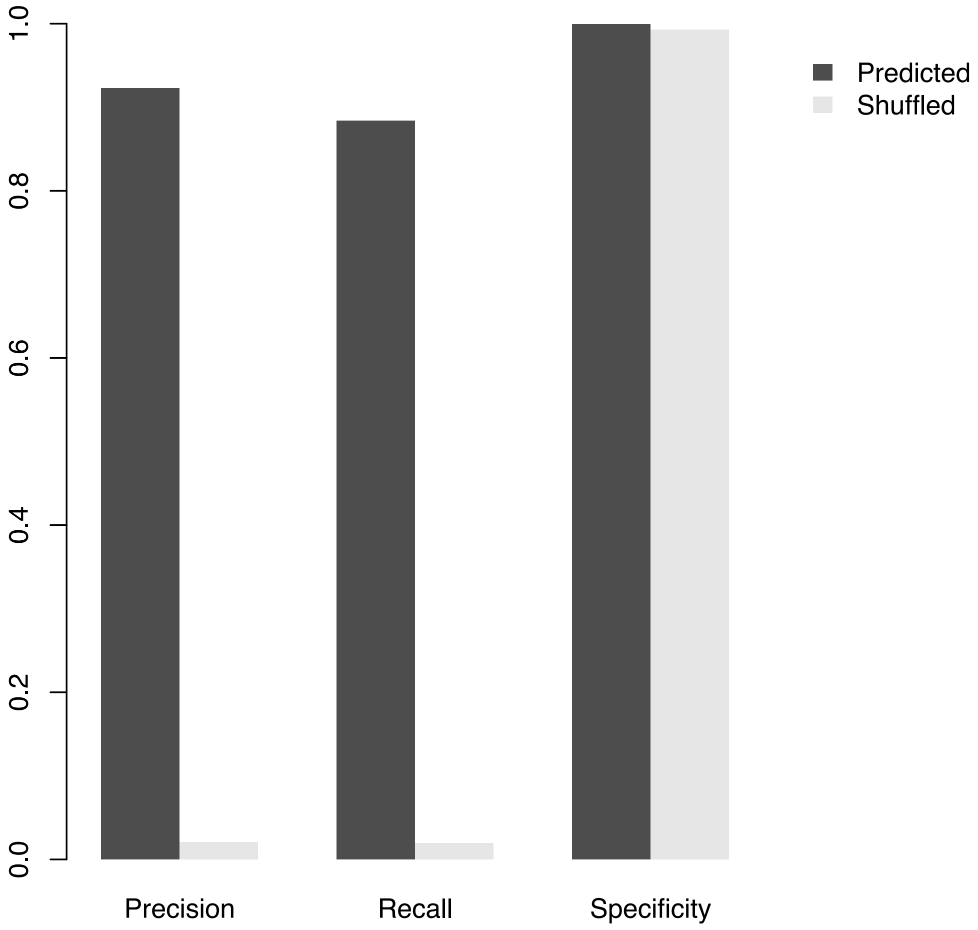

Supplementary Fig. 17. Performance of patient association to their corresponding disease (black bars) against random expectation (grey bars). Random expectation is generated shuffling patients and calculating Euclidean distances to all of them, associating each patient to the disease of the most similar patient.

Supplementary tables

Supplementary Table 1. Number of positive interactions between diseases (coded in ICD10), their overlap with epidemiology and the significance of such overlap extracted from microbiome, miRNA and GWAS-based disease networks.

Omic	pval	intersection	total	epidemiology
Microbiome	0.3707	6	47	6
miRNA	0.59294	16	519	18
GWAS	0.07189	13	375	22

Supplementary Table 2. Intra-disease interaction percentages.

Disease	%	Disease	%	Disease	%
Acne	100	Glioblastoma	98,18	Pancreatic ductal adenocarcinoma	76,12
Acute atopic dermatitis	100	Familial hemophagocytic lymphohistiocytosis	98,18	Hepatitis C virus infection	76,03
Adult T cell leukemia	100	T cell lymphoma	98,06	Thalassemia	75
Alcoholic hepatitis	100	Atrial fibrillation	97,8	Lobular breast cancer	71,42
Alopecia	100	Turner syndrome	96,92	Coenzyme Q deficiency	71,21
Arrhythmogenic right ventricular cardiomyopathy	100	Dermatomyositis	96,54	Sybsyndromal symptomatic depression	70,51
Autosomal dominant monocytopenia	100	Idiopathic pulmonary fibrosis	96,32	Ductal breast cancer	70
Basaloid lung cancer	100	Hepatocellular carcinoma	95,55	Sotos syndrome	69,28
Campylobacter jenuni infection	100	Astrocytoma	94,95	Crohns disease	69,23
Chronic lymphocytic leukemia	100	Uremia	94,12	Cornelia de Lange syndrome	69,11
Cutaneous sarcoidosis	100	Cardioembolic stroke	94,03	Myeloma	67,85
Ependymoma	100	ANCA crescentic glomerulonephritis	93,33	Myelodysplastic syndrome	65,39
Esophageal squamous cell carcinoma	100	Primary myelofibrosis	93,33	Human papillomavirus cervix	56,09
Fatal familial insomnia	100	Dengue	91,91	Polycystic ovary syndrome	54,16
Follicular lymphoma	100	Vitiligo	91,49	Sickle cell anemia	52,38
Hepatocellular carcinoma associated to hepatitis B	100	Systemic lupus erythematosus	90,98	Aphthous stomatitis	50,54
Hutchinson Gilford progeria	100	Thyroid cancer	90,97	Spina bifida	50
Interstitial cystitis	100	Polycythemia vera	90,97	Peripheral arterial disease	49,7
Ischemic heart	100	Cervical cancer	90,7	Amyotrophic lateral sclerosis	49,4
ISCU myopathy	100	Colorectal cancer	90,56	Acute myeloid leukemia	47,67
Job's syndrome	100	Septic shock	90,22	Acute myocardial infarction	46,34
Klinefelter's syndrome	100	NSCLC	89,96	Ageing	45,87
Leishmania infection	100	Atopic dermatitis	89,66	Thrombocytopenia	44,44
Lung squamous cell carcinoma	100	Training	89,39	Sarcoidosis	43,79
Medulloblastoma	100	Chronic atopic dermatitis	89,28	Parkinson's disease	43,3
Multiple myeloma	100	Myositis	88,81	Axial spondyloarthropathy	38,56
Mycobacterium tuberculosis	100	Malignant pleural mesothelioma	88,68	Myocardial infarction	37,84
Osteosarcoma	100	Trachoma	87,56	Choroideremia	33,33
Pancreatic carcinoma	100	Renal cancer	86,66	Type I diabetes	32,08
Pilocytic astrocytoma	100	Prostate cancer	85,46	Smoker	28,66
Rhabdoid tumor	100	Oligodendroglioma	84,8	Ischemic stroke	26,84
Erythematotelangiectatic Rosacea	100	Juvenile myelomonocytic leukaemia	84,49	Behcet's disease	26,66
Papulopustular Rosacea	100	Leigh syndrome	84,44	Huntington's disease	26,14
Phymatous Rosacea	100	Interstitial lung disease	84,18	Sjogren's syndrome	26,13
Seborrheic keratosis	100	Chronic rhinosinusitis	83,51	Irritable bowel syndrome	24,67
Setleis syndrome	100	Essential thrombocythemia	83,04	Alzheimer's disease	23,8
Smoldering myeloma	100	Monoclonal gammopathy	82,87	Endometriosis	22,84
Muscular dystrophy	100	Psoriasis	81,8	Type II diabetes	22,64
Ovary cancer	100	Osteoarthritis	80	Gastric cancer	19,7
Lung cancer	100	Pollen allergy	80	Multiple sclerosis	19,68
Hepatoblastoma	100	Mitochondrial DNA polymerase POLG1 disease	80	Asthma	15,51
Nasopharingeal carcinoma	100	Breast cancer	79,29	Autism	15,34
Bladder carcinoma	99	Oral cavity cancer	77,39	Chronic obstructive pulmonary disease	10,29
Oral dysplasia	99	Adrenocortical carcinoma	77,34	Schizophrenia	8,86
Hepatitis B	98	Ulcerative colitis	76,62	Major depression	7,53

Supplementary Table 3. Summary statistics of the overlaps between our molecular networks (Fisher's exact tests selecting different numbers of genes, correlations and meta-analyses) with epidemiological networks (Hidalgo *et al.* [23] in ICD9 and Jensen *et al.* [3] in ICD10).

	ICD9								ICD10							
	positive interactions				negative interactions			epidemiology	positive interactions				negative interactions			epidemiology
Genes Selected	pval	intersection	total	%	pval	intersection	total	total	pval	intersection	total	%	pval	intersection	total	total
100 genes	0	215	370	6,3	1	53	110	3412	0.00194	12	275	13	0.99122	1	103	95
200 genes	0	343	649	10	1	123	289	3412	0.00611	15	446	16	0.98225	3	242	95
300 genes	1,00E-05	451	958	13	1	235	561	3412	0.00134	22	670	23	0.88565	8	479	95
400 genes	0	439	876	13	1	196	471	3412	0.00577	19	618	20	0.82275	8	409	95
500 genes	0.00885	541	1233	16	1	364	922	3412	0.00869	24	881	25	0.72997	13	747	95
1000 genes*	0.06096	687	1617	20	0.99999	586	1559	3412	0.01231	29	1166	31	0.77109	17	1226	95
1500 genes*	0.12266	732	1743	21	0.93684	683	1792	3412	0.04711	28	1242	29	0.32046	23	1388	95
2000 genes	0.30773	763	1848	22	0.51308	753	2017	3412	0.19935	26	1327	27	0.45258	23	1526	95
*2500 genes	0.04266	819	1929	24	0.41048	791	2131	3412	0.15504	28	1399	29	0.57128	23	1582	95
*3000 genes	0.01087	867	2018	25	0.174	841	2250	3412	0.35971	26	1444	27	0.63711	23	1645	95
3500 genes	0.01804	878	2054	26	0.03387	873	2340	3412	0.24683	28	1477	29	0.68729	23	1717	95
4000 genes	0.02202	886	2077	26	0.01349	890	2416	3412	0.29921	28	1519	29	0.56637	25	1759	95
4500 genes	0.00929	908	2114	27	0.00818	909	2476	3412	0.3417	28	1548	29	0.4301	27	1770	95
5000 genes	0.00419	929	2151	27	0.01912	918	2512	3412	0.27862	29	1562	31	0.54067	26	1789	95
Correlations	0.00994	1058	2478	31	0	1109	2879	3412	0.07181	38	1849	40	0.6096	30	1998	95
Meta-analysis	0	177	405	20	0.99642	62	160	892	-	-	-	-	-	-	-	-

Supplementary References

1. Buniello, A. *et al.* The NHGRI-EBI GWAS Catalog of published genome-wide association studies, targeted arrays and summary statistics 2019. *Nucleic Acids Res.* **47**, D1005–D1012 (2019).
2. Bodenreider, O. The Unified Medical Language System (UMLS): Integrating biomedical terminology. *Nucleic Acids Res.* **32**, D267-70 (2004).
3. Jensen, A. B. *et al.* Temporal disease trajectories condensed from population-wide registry data covering 6.2 million patients. *Nat. Commun.* **5**, 4022 (2014).
4. Zhou, X., Menche, J., Barabási, A.-L. & Sharma, A. Human symptoms–disease network. *Nat. Commun.* **5**, 4212 (2014).
5. Bodenreider, O. The Unified Medical Language System (UMLS): Integrating biomedical terminology. *Nucleic Acids Res.* **32**, (2004).
6. Serrano, D. *et al.* Inhibition of telomerase activity preferentially targets aldehyde dehydrogenase-positive cancer stem-like cells in lung cancer. *Mol. Cancer* **10**, 96 (2011).
7. Liu, M. *et al.* Telomere Shortening in Alzheimer's Disease Patients. *Ann. Clin. Lab. Sci.* **46**, 260–5 (2016).
8. Weaver, D. F. *et al.* ALZHEIMER'S DISEASE AS A DISORDER OF TRYPTOPHAN METABOLISM. *Alzheimer's Dement.* **13**, P1267 (2017).
9. Juhász, C. *et al.* Quantification of tryptophan transport and metabolism in lung tumors using PET. *J. Nucl. Med.* **50**, 356–63 (2009).
10. Chuang, S.-C. *et al.* Circulating Biomarkers of Tryptophan and the Kynurenine Pathway and Lung Cancer Risk. *Cancer Epidemiol. Biomarkers Prev.* **23**, 461–468 (2014).
11. Chen, M.-H. *et al.* Risk of Dementia Among Patients With Asthma: A

Nationwide Longitudinal Study. *J. Am. Med. Dir. Assoc.* **15**, 763–767 (2014).

12. Liao, K.-M., Ho, C.-H., Ko, S.-C. & Li, C.-Y. Increased Risk of Dementia in Patients With Chronic Obstructive Pulmonary Disease. *Medicine (Baltimore).* **94**, e930 (2015).

13. Li, X., Song, D. & Leng, S. X. Link between type 2 diabetes and Alzheimer's disease: from epidemiology to mechanism and treatment. *Clin. Interv. Aging* **10**, 549–60 (2015).

14. Cai, L. & Huang, J. Schizophrenia and risk of dementia: a meta-analysis study. *Neuropsychiatr. Dis. Treat.* **14**, 2047–2055 (2018).

15. Hu, J., Klein, J. D., Du, J. & Wang, X. H. Cardiac muscle protein catabolism in diabetes mellitus: activation of the ubiquitin-proteasome system by insulin deficiency. *Endocrinology* **149**, 5384–90 (2008).

16. Simpson, J. L., Scott, R. J., Boyle, M. J. & Gibson, P. G. Differential Proteolytic Enzyme Activity in Eosinophilic and Neutrophilic Asthma. *Am. J. Respir. Crit. Care Med.* **172**, 559–565 (2005).

17. Jevnikar, Z. *et al.* Epithelial IL-6 trans-signaling defines a new asthma phenotype with increased airway inflammation. *J. Allergy Clin. Immunol.* (2018). doi:10.1016/j.jaci.2018.05.026

18. Giebelstein, J. *et al.* The proteomic signature of insulin-resistant human skeletal muscle reveals increased glycolytic and decreased mitochondrial enzymes. *Diabetologia* **55**, 1114–1127 (2012).

19. Matheson, M. C. *et al.* β2-adrenergic receptor polymorphisms are associated with asthma and COPD in adults. *J. Hum. Genet.* **51**, 943–951 (2006).

20. Maletic, V., Eramo, A., Gwin, K., Offord, S. J. & Duffy, R. A. The Role of Norepinephrine and Its α-Adrenergic Receptors in the Pathophysiology and

Treatment of Major Depressive Disorder and Schizophrenia: A Systematic Review. *Front. Psychiatry* **8**, 42 (2017).

21. Hu, J. *et al.* CNTN6 copy number variations in 14 patients: a possible candidate gene for neurodevelopmental and neuropsychiatric disorders. *J. Neurodev. Disord.* **7**, 26 (2015).

22. Ghani, M. *et al.* Genome-Wide Survey of Large Rare Copy Number Variants in Alzheimer's Disease Among Caribbean Hispanics. *G3: Genes|Genomes|Genetics* **2**, 71–78 (2012).

23. Hidalgo, C. A., Blumm, N., Barabási, A.-L. & Christakis, N. A. A Dynamic Network Approach for the Study of Human Phenotypes. *PLoS Comput. Biol.* **5**, e1000353 (2009).

24. Ma, W. *et al.* An analysis of human microbe–disease associations. *Brief. Bioinform.* **18**, 85–97 (2017).

25. Lu, M. *et al.* An analysis of human microRNA and disease associations. *PLoS One* **3**, e3420 (2008).

26. Shannon, P. *et al.* Cytoscape: A Software Environment for Integrated Models of Biomolecular Interaction Networks. *Genome Res.* **13**, 2498–2504 (2003).
