# Supplementary material for: Interpreting molecular similarity between patients as a determinant of disease comorbidity relationships
Source: Nat Commun. 2020 Jun 5;11:2854. doi: 10.1038/s41467-020-16540-x (PMC7275044; doi:10.1038/s41467-020-16540-x)
Supplement: Supplementary file 2 — Description of Additional Supplementary Files [file 41467_2020_16540_MOESM2_ESM.pdf]

Description of Additional Supplementary Files

File Name: Supplementary Data 1
Description: Number of interactions between ICD9 categories observed in the Phenotypic Disease Network (PMID:19360091).

File Name: Supplementary Data 2
Description: Number of interactions between ICD10 categories observed in the Temporal Disease Trajectories (PMID:24959948).

File Name: Supplementary Data 3
Description: Coordinately deregulated genes in each molecularly homogeneous patient-subgroup. “0”, “-“, and “+” denote that the gene is either not coordinately deregulated, coordinately down-regulated or coordinately up-regulated in the patient-subgroup.

File Name: Supplementary Data 4
Description: Datasets analyzed during the study, with the number of associated case and control samples.
